# Niche differentiation of sulfur-oxidizing bacteria (SUP05) in submarine hydrothermal plumes

**DOI:** 10.1038/s41396-022-01195-x

**Published:** 2022-01-26

**Authors:** Bledina Dede, Christian T. Hansen, Rene Neuholz, Bernhard Schnetger, Charlotte Kleint, Sharon Walker, Wolfgang Bach, Rudolf Amann, Anke Meyerdierks

**Affiliations:** 1 Max Planck Institute for Marine Microbiology, Bremen, Germany; 2 MARUM, Center for Marine Environmental Sciences, University of Bremen, Bremen, Germany; 3 Institute for Chemistry and Biology of the Marine Environment (ICBM), Carl von Ossietzky University of Oldenburg, Oldenburg, Germany; 4 Department of Physics and Earth Sciences, Jacobs University Bremen, Bremen, Germany; 5 National Oceanic and Atmospheric Administration, Pacific Marine Environmental Laboratory, Seattle, WA USA; 6 Geoscience Department, University of Bremen, Bremen, Germany; 7 Present Address: Fraunhofer Institute for Manufacturing Technology and Advanced Materials (IFAM), Group: Quality Assurance and Cyber-Physical Systems, Bremen, Germany

**Keywords:** Biodiversity, Molecular ecology, Metagenomics, Microbial ecology

## Abstract

Hydrothermal plumes transport reduced chemical species and metals into the open ocean. Despite their considerable spatial scale and impact on biogeochemical cycles, niche differentiation of abundant microbial clades is poorly understood. Here, we analyzed the microbial ecology of two bathy- (Brothers volcano; BrV-cone and northwest caldera; NWC) and a mesopelagic (Macauley volcano; McV) plumes on the Kermadec intra-oceanic arc in the South Pacific Ocean. The microbial community structure, determined by a combination of 16S rRNA gene, fluorescence in situ hybridization and metagenome analysis, was similar to the communities observed in other sulfur-rich plumes. This includes a dominance of the vent characteristic SUP05 clade (up to 22% in McV and 51% in BrV). In each of the three plumes analyzed, the community was dominated by a different yet uncultivated chemoautotrophic SUP05 species, here, provisionally named, *Candidatus* Thioglobus vadi (McV), *Candidatus* Thioglobus vulcanius (BrV-cone) and *Candidatus* Thioglobus plumae (BrV-NWC). Statistical analyses, genomic potential and mRNA expression profiles suggested a SUP05 niche partitioning based on sulfide and iron concentration as well as water depth. A fourth SUP05 species was present at low frequency throughout investigated plume samples and may be capable of heterotrophic or mixotrophic growth. Taken together, we propose that small variations in environmental parameters and depth drive SUP05 niche partitioning in hydrothermal plumes.

## Introduction

Hydrothermal vents occur along oceanic spreading zones and volcanic arcs, in back-arc basins, and in intra-plate volcanoes. At these sites, high-temperature fluids enriched in reduced chemical compounds vent from the seafloor and mix with ambient seawater until they reach a depth of neutral buoyancy. These plumes spread over large spatial scales and thus, have a substantial impact on biogeochemical cycles [1–4]. As the vent-sourced catabolic energy input is considerable for the deep-sea [5], plumes offer a thriving habitat for microorganisms. Microbial communities inhabiting hydrothermal plumes are diverse, owing to the mixing of typical deep-sea bacteria, such as SAR11, SAR324 and MG-I Archaea [6] with chemolithoautotrophs indicative of the different physico-chemical plume signatures [7, 8].

A microbial clade well known to inhabit hydrothermal sulfur-rich plumes, is the SUP05 clade within the Gammaproteobacteria [9]. Members of this diverse clade have successfully adapted to various lifestyles such as free-living organisms in plumes [8], oxygen-minimum zones (OMZ) [10–12], pelagic redoxclines [13, 14] and as symbionts of clams, mussels and sponges [15–17]. In addition to dark carbon fixation fueled by reduced sulfur compounds and hydrogen oxidation [18], SUP05 clade bacteria have also been postulated to maintain a heterotrophic metabolism [19, 20]. Several representatives of this clade have already been cultivated and assigned to the genera *Candidatus* Thioglobus and Pseudothioglobus [21–23]. Despite the widespread occurrence and high diversity of this clade, the localization and niche partitioning of SUP05 in hydrothermal plumes has not yet been elucidated.

In this study, we address microbial diversity and niche partitioning of SUP05 in hydrothermal plumes derived from degassing volcanoes in the Kermadec intra-oceanic arc. The vent fluids in this hydrothermal system exhibit large compositional variability due to differences in the type of sub-seafloor magmatic-hydrothermal reactions and water depth [24, 25]. We investigated three plumes in the Kermadec Arc, one of which is sourced from Macauley volcano (McV, ~300 m depth) and the other two are sourced from two distinct hydrothermal sites hosted in the Brothers volcano (BrV, ~1600 m depth). Kleint et al. [26] have shown that the geochemical variability of these vent fluids in terms of acidity as well as metal and gas contents is extremely large. In our study, we combined an extensive geochemical dataset with 16S rRNA gene analysis, fluorescence in situ hybridization (FISH), metagenomics and metatranscriptomics to distinguish different species within the SUP05 clade and develop a hypothesis on SUP05 niche separation.

## Methods

### Site description and sampling

The Kermadec Arc is one of the most hydrothermally active intra-oceanic arcs in the world [24, 25]. Two of its submarine volcanoes, McV and BrV were sampled during R/V Sonne expedition SO253 in December 2016–January 2017. McV rises to a depth of ~300 m below sea level (mbsl) and emits fluids influenced by SO_2_-rich magmatic vapors. Disproportionation of SO_2_ upon cooling results in high concentrations of H_2_S (10 mM) and sulfuric acid, the dissociation of which causes low pH and high contents of total dissolved iron (DFe) (1.7 mM) [26]. BrV, in contrast, exhibits vents with different fluid compositions: (1) NW Caldera (NWC) site (~1600 mbsl) is influenced by water-rock interaction and shows high DFe (12.4 mM) and low H_2_S (1.1 mM); (2) two resurgent volcanic edifices in the SE section of the caldera—the upper cone (1220 mbsl) and lower cone (1320 mbsl) hydrothermal sites—are of magmatic-hydrothermal origin with high H_2_S (5.0 mM) and low metal (DFe: 15 µM) concentrations [26].

Hydrothermal plumes were mapped and discrete water samples were collected using a conductivity-temperature-depth device (CTD) during vertical casts and tow-yo operations [27]. The Seabird 9*plus* CTD had integrated sensors for optical backscatter (Seapoint Turbidity Meter, 5X custom sensitivity) and oxidation-reduction potential (NOAA-PMEL ORP sensor; [28]) in a rosette with 22 10-L Niskin bottles.

Water samples were filtered through 0.22 μm pore size polycarbonate membrane filters directly after retrieval of the CTD on board. For FISH, cells were fixed with formaldehyde prior to filtration (details on sample collection: Supplementary Materials and Methods).

### 16S rRNA gene analysis and CARD-FISH

Genomic DNA was extracted using the PowerSoil DNA Isolation Kit (MoBio, Ca, USA). The V3-V4 region of the 16S rRNA gene was amplified using the primer combination Bakt_341F and Bakt_805R [29] with barcodes [30]. PCR products were sequenced on an Ion Torrent Personal Genome Machine (PGM) System (Thermo Fisher Scientific, Waltham, MA USA) using Ion PGM Hi-Q chemistry, partly on-board (details: Supplementary Materials and Methods).

Adapters were trimmed with cutadapt v1.9 [31]. Mothur v1.39.5 [32] was used for quality trimming (qthreshold = 10, minlength = 250 bp and maxhomop = 10). In total, 20,000 reads were subsampled using the reformat.sh script from BBTools v35.14 [33]. Subsampled reads were analyzed using the SilvaNGS v1.3 pipeline [34] with the SILVA SSU Ref taxonomy (release 132) and default parameters. Phylogenetic trees were constructed using the software package ARB [35] (details on phylogenetic tree reconstruction: Supplementary Materials and Methods).

Catalyzed reporter deposition-FISH (CARD-FISH) analysis was performed according to Pernthaler et al. [36]. CARD-FISH details, including probes used in this study and counting details are described in Supplementary Materials and Methods.

### Metagenome sequence and analysis

Genomic DNA was sheered to a fragment size of ~400 bp using a S2 sonicator (Covaris, Woburn, MA USA) followed by the protocol “Ovation Ultralow System v2 1-16” (NuGEN, CA, USA). The fragments were sequenced in paired-end mode (2 × 250 bp) on a HiSeq 2500 (Illumina, San Diego, CA, USA) at the Max Planck Genome Centre (Cologne, Germany).

Metagenomic reads were first quality checked with FastQC v0.11.7 [37]. BBDuk part of BBTools v35.14 [33] was used for read trimming with minimum quality Phred score of 20 and 100 bp minimum length. Additionally, Nonpareil was used to assess the level of coverage in our metagenomes [38].

Subsequently, metagenomes were assembled with MEGAHIT v1.0.2 [39] using k-mer steps of 10 and a maximum k-mer size of 127. In order to retrieve high completeness and low contamination MAGs, each metagenome was individually assembled and co-assembled with other metagenomes coming from the same hydrothermal system (BrV or McV).

Individual assemblies of five McV metagenomes were binned with Metawatt v3.5.3 [40] (default parameters, contig threshold = 1 kb, minimum seed bin size = 5 kb). Four single assemblies of BrV metagenomes were binned using CONCOCT [41] (default parameters) in anvi’o v6.1 [42]. Additionally, a co-assembly of the four metagenomes from BrV resulted in MAGs with higher completeness and less contamination compared to individual assemblies of the same metagenomes. Moreover, targeted re-assembly, as described in Meier et al. [43], was performed on Gammaproteobacteria MAGs using the SPAdes assembler v3.10.1 [44] (default parameters, careful mode). After the application of 2–3 re-assembly rounds, twelve more than 50% complete SUP05-related MAGs were retrieved. Completeness and contamination of the MAGs was analyzed by CheckM [45]. All MAGs were visualized and manually refined using anvi’o v6.1 [42]. For details on MAG analysis see Supplementary Materials and Methods.

### Metatranscriptome sequence and analysis

Filter pieces were thawn on ice and RNAlater was removed carefully (exception: 54CTD_b12). Total RNA was extracted using a phenol/chloroform based extraction method with polyvinylpolypyrrolidone containing extraction buffer (for details see Supplementary Materials and Methods). DNA was removed by DNase treatment (Turbo DNase, Thermo Fisher Scientific) and total RNA was purified using RNA clean and concentrator (Zymo Research, Irvine, CA, USA). Capillary electrophoresis (Picochip, Agilent Bioanalyser; Agilent, Santa Clara, CA, USA) was used for quality assessment. An Illumina-compatible library was generated with the NEBNext Ultra II Directional RNA Library Prep kit (NEB, Ipswich, MA, USA) followed by sequencing on a HiSeq 2500 platform (2 × 250 bp; Illumina).

Reads were quality trimmed using Trimmomatic v0.39 [46] with phred 33, minimum length of 70 bp and a sliding window every 4 bp for a required quality 15. After sorting of rRNA reads using SortMeRNA [47] 16S rRNA sequences were classified using the SilvaNGS pipeline [34] with SILVA SSU138 taxonomy. Metatranscriptomes were mapped onto annotated SUP05-related MAGs (minid = 97) using BBMap [33] and reads mapped were normalized using Geneious Prime 2019.2 (Biomatters, Auckland, New Zealand). Transcripts per million (TPMs) were calculated for normalization of transcripts. The expression of house-keeping genes was estimated by calculating the TPM of three reference genes (*proC*, *recA*, *rpoD*) [48] that were present in all MAGs. The heatmap was created with pheatmap [49] and the viridis [50] package in R [51]. Additionally, metagenome and metatranscriptome raw reads were mapped to SUP05-related MAGs using BBMap (minid = 99%) [33]. In order to account for ambiguity, metatranscriptomes and metagenomes were mapped to MAGs sharing an ANI > 98% using BBSplit of the BBTools package (minid = 99%) [33]. RPKM was calculated based on MAG length and number of reads mapped.

### Thermodynamic calculations

Gibb’s free energy (∆_r_G) per mol of substrate was calculated using procedures detailed in Meier et al. [43]. We used Geochemist’s Workbench (Aqueous Solutions LLC, Champaign, IL) and a 250 bar thermodynamic database to compute reference state Gibb’s free energies (∆_r_G^o^) and activity coefficients. Measured DFe concentration in plume waters were used to compute ∆_r_G (details: Supplementary Materials and Methods section).

### Statistical analysis

Statistical analyses were carried out in R [51] using the vegan [52], stats v3.6.2 and gvlma v1.0.0.3 [53] packages. Multivariate analyses (Redundancy, perMANOVA; ref. [54], and linear regression) were performed using a Bray–Curtis dissimilarity matrix with a 1% abundance threshold and standardized (log transformed), geochemical parameters (details: Supplementary Materials and Methods).

### Data accession

Raw reads and Metagenome-Assembled Genomes (MAGs) were submitted to the European Nucleotide Archive under project number PRJEB42974. Metagenomes are found under the accession numbers SAMEA8000741–SAMEA8000749, metatranscriptomes are found under SAMEA8000750–SAMEA8000753. 16S rRNA gene amplicons can be found under project accession PRJNA721877.

## Results

### Microbial diversity

**Fig. 1:**
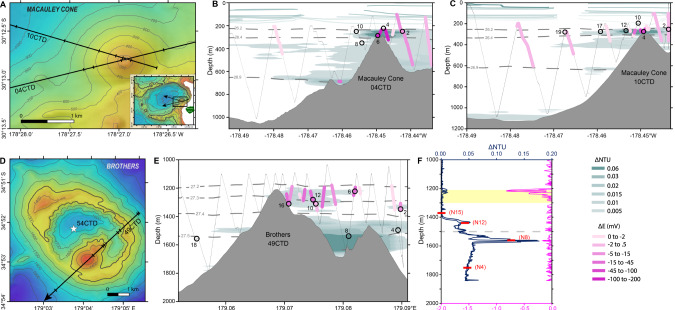
Sample locations and hydrothermal plume distributions defined by turbidity (∆NTU) and oxidation-reduction potential anomalies (∆E or dE/dt) during CTD operations. For tow transects, thin dotted lines represent tow tracklines; thick dashed lines are potential density contours; Niskin bottle samples are indicated by open circles with the bottle number adjacent to each symbol. Niskin bottle samples for the vertical profile are indicated by red bars, with the bottle number adjacent to each symbol. **A** Map showing CTD tow tracklines at McV cone. Inset shows the entire McV (arrows in inset show direction and total length of each tow). **B** Plume distribution and sample locations during tow-yo 04CTD. **C** Plume distribution and sample locations during tow-yo 10CTD. **D** Map of CTD tow-yo trackline (arrow shows direction and length of tow) and vertical profile (star) at BrV. **E** Plume distribution and sample locations during tow-yo 49CTD. Note two dominant plume layers detected at Brothers are emitted from the NW Caldera Wall (deeper than ~1400 m) and cone (Upper and Lower) sites (~1200–1350 m). **F** Turbidity and oxidation-reduction potential (ORP) anomaly (as dE/dt) profiles of vertical cast 54CTD at BrV. The caldera rim depth is shown by a gray dashed line at 1500 m. The (ORP) anomaly is only associated with the cone plume depth. A light yellow rectangle indicates the depth range of the cone summit depths and cone-sourced plumes.

In order to explore the structure and function of McV and BrV plume microbial communities, 26 samples were collected with tow-yo and vertical CTD casts at different depths, chemical properties and plume intensities (Table S1). The dataset comprises two background samples from outside of the main hydrothermal fields at comparable depths, 11 plume samples taken at McV (depth between 195 mbsl and 351 mbsl) and 13 plume samples from BrV (depth between 1229 mbsl and 1750 mbsl) (Fig. 1).

**Fig. 2:**
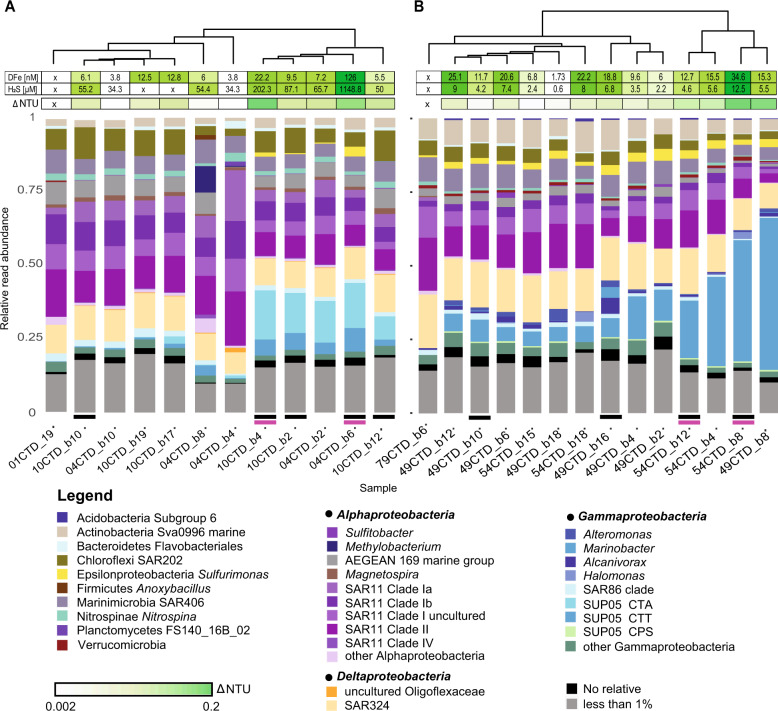
Relative read abundance of 16S rRNA gene amplicon sequences. The dendrogram in the upper part of the graph depicts the result of a complete linkage hierarchical clustering based on a Bray–Curtis dissimilarity matrix of the community composition at (**A**) McV and (**B**) BrV. Distribution of turbidity (∆NTU) is given below the hierarchical clustering indicating the intensity of the plume. The taxonomic assignment is based on the SilvaNGS pipeline [34] using SILVA SSU132 taxonomy. SUP05 subgroups were distinguished based on phylogenetic analysis using ARB. Taxonomic groups with <1% read abundance were combined for phylum level read abundance. SUP05 sequences are related to CTA *Candidatus* Thioglobus autotrophicus, CTT *Candidatus* Thioglobus thermophilus, CPS *Candidatus* Pseudothioglobus singularis. Metagenome samples are indicated with a black line underneath the stacked barchart and metatranscriptomes are indicated with a pink line.

Microbial communities were dominated by clades within the Alphaproteobacteria, Deltaproteobacteria (reclassified as Deltabacterota phy. nov.; ref. [55]) and Gammaproteobacteria. At a higher taxonomic resolution, the most prevalent taxa included different subclades of the SAR11 (Alphaproteobacteria) (up to 60% in McV and 25% in BrV), the SAR324 phylum (up to 13% in McV and 16% in BrV) and the gammaproteobacterial SUP05 clade (1–23% in McV and 5–51% in BrV) (Fig. 2). Within the SUP05, different subclades dominated at McV compared to BrV. Phylogenetic analysis (Fig. S1) classified these as *Candidatus* Thioglobus autotrophicus-related (CTA), *Candidatus* Thioglobus thermophilus-related (CTT) and *Candidatus* Pseudothioglobus singularis-related (CPS) bacteria. CTA sequences were present exclusively at McV (up to 17%). CPS sequences were less than 2% abundant across all samples at BrV and <1% at McV. CTT sequences ranged up to 8% in McV and dominated the BrV SUP05 community (up to 49%). Vent characteristic *Sulfurimonas* were present in all samples, constituting up to 4% read abundance.

### Statistical analysis and thermodynamic considerations

Distance based redundancy analysis (dbRDA) indicated a correlation between microbial community composition and changes in geochemical parameters (Fig. S2). Firstly, dbRDA revealed that, at McV, CTA were shown to be positively impacted by DFe, temperature, DOC, PO_4_ and TDN. A PerMANOVA test confirmed that the parameters that could explain the community the most were O_2_ (53% *p* = 0.001) and DFe (13% *p* = 0.002). On the contrary, at BrV, CTT was negatively impacted by DOC, temperature and O_2_. At BrV, a PerMANOVA indicated that DFe (20% *p* = 0.08) was the only significant parameter.

Furthermore, a linear regression was applied in order to model the relationship between the microbial community and the geochemical parameters (Table S2). At McV, the relative abundance of CTA was positively affected by decreasing H_2_S concentration (*p* = 0.02) and increasing DFe (*p* = 0.02), total dissolved oxygen (O_2_) (*p* = 0.003) and temperature (*p* = 0.05). Whereas, CTT present in all BrV samples, responded positively to higher PO_4_ (*p* = 0.01), depth (*p* = 0.07) and lower TDN (*p* = 0.01), CPS was significantly affected only by pH (*p* = 0.01).

Thermodynamic calculations indicated that aerobic oxidation of sulfide was the most energetically favorable process along all sites (maximum values: McV: −97.38 kj/mol e^−^; BrV: −96.37 kj/mol e^−^), releasing almost twice as much kJ per mol electron than iron oxidation (maximum values: McV: −58.85 kj/mol e^−^; BrV: −56.16 kj/mol e^−^) (Fig. S3).

### Microbial abundance determined by CARD-FISH

CARD-FISH was conducted on representative samples to confirm the relative abundance patterns of Bacteria, Archaea, Gammaproteobacteria, and specifically, SUP05-clade bacteria (Fig. S4).

At McV total cell counts were in a range from 9.4 × 10^4^ to 1.5 × 10^5^ cells/ml. Bacteria accounted for 29% (04CTD_b6) – 52% (10CTD_b2) of all cells, dominating over Archaea with 20% (04CTD_b6) – 25% (04CTD_b4). CARD-FISH counts confirmed a high abundance of SUP05-clade bacteria at McV with up to 24% in the samples with a high turbidity (∆NTU). The total number of other Gammaproteobacteria, was overall in a range of 0.7% (10CTD_b10) to 6% (04CTD_b10).

At BrV, total cell counts ranged between 2.7 × 10^4^ to 4.3 × 10^4^ cells/ml. Also here, Bacteria dominated (52% 54CTD_b15 – 80% 54CTD_b8) over Archaea (8% 54CTD_b8 – 19% 54CTD_b15). As already suggested by 16S rRNA gene amplicon analysis, samples characterized by a high ∆NTU contained an increased abundance of SUP05-related bacteria (up to 67% 54CTD_b8). Other Gammaproteobacteria were present in low abundance in a range from 0.2% (54CTD-b4) to 5% (49CTD_b2).

Additionally, all samples were checked for the presence of grazers by DAPI staining, yet no evidence for eukaryotes with food vacuoles was found.

### Metagenomes analysis

Nine representative samples were selected for metagenomic sequencing in order to elucidate the metabolic potential of abundant clades, these included five samples from McV (04CTD_b6, 10CTD_b2, 10CTD_b4, 10CTD_b10 and 10CTD_b12) and four from BrV (cone: 49CTD_b10, 49CTD_b16; NWC: 54CTD_b8 and 54CTD_b12) (Fig. 1 and Table S3).

According to Nonpareil [38], the sequencing depth of the metagenomes covered 41–72% of the community (Table S3). The taxonomic distribution of McV and BrV metagenomes was analyzed by sorting and classifying the 16S rRNA marker gene using SortMeRNA [47]. This largely supported the 16S rRNA gene amplicon analysis with a fluctuation of up to 7% for SUP05 subgroups (Fig. S5).

### Community-wide functional analysis between plumes

In order to compare the functional capacities between McV and BrV as well as other plumes, a community-wide analysis was conducted based on functional genes. The other samples analyzed were Woody Crack (buoyant plume, Menez Gwen, Mid Atlantic Ridge) [43], Mariner and Kilo Moana (plume, Eastern Lau Spreading Centre (ELSC)) [56] and background South Pacific Ocean samples [57]. Based on a dissimilarity matrix of functional genes, a neighbor joining tree revealed five distinct groups: (1) McV, (2) BrV, (3) ELSC, (4) Woody Crack metagenomes and (5) background samples, reflecting their diverse geochemical properties and/or the geographical distances between them (Fig. S6). According to a bi-plot PCoA analysis, the variable contributing the most to the dissimilarities was the relative abundance of the cytochrome *c* oxidase and cytochrome *b* gene. This was supported by a high number of cytochrome *c* oxidase and cytochrome *b* genes in McV metagenomes (Fig. S7). SUP05-affiliated cytochrome genes reached up to 34% of all cytochromes at McV and up to 23% of all cytochromes at BrV (Fig. S8).

### Metagenome-assembled genomes

A total of 17 MAGs were retrieved (Table S4), that past a completeness threshold of 50%, and were taxonomically affiliated with *Sulfurimonas*, uncultured Chloroflexi, uncultured Acidimicrobiales, SAR324, *Erythrobacter* (Fig. S9) and the SUP05 clade.

MAG-1 to -4 were obtained from McV and have completeness values of 70–78% (contamination 4–10%). Six SUP05 MAGs (MAG-5_1, MAG-6_1, MAG-6_2 and MAG-7_1 to MAG-7_3) from BrV had completeness values ranging from 53% (MAG-7_2) to 86% (MAG-5_1). Additionally, a cross-assembly of BrV metagenomes resulted in higher completeness SUP05 MAGs: MAG-5 (completeness 94%, contamination 0%) and MAG-6 (completeness 92%, contamination 0.8%). MAGs were subsequently de-replicated at a 95% ANI threshold (65% coverage threshold), generating three species clusters: SUP05–1–4 (MAG-1 to 4), SUP05–5 (MAG-5 and 5_1) and SUP05–6 (MAG-6, 6_1 and 6_2) (Table S5).

SUP05-1-4 had a high abundance in the plume of McV (04CTD_b6, 10CTD_b2, 10CTD_b4 and 10CTD_b12) and were almost absent from BrV plumes. In contrast, SUP05–5 and -6 prevailed in plumes at BrV and were rare in the McV plume. SUP05-5 was ~3.8 fold more abundant in the BrV-Cone plume compared to SUP05-6, but SUP05-6 was ~1.7 fold more abundant in the BrV-NWC than SUP05-5 (Table S4). Physico-chemical parameters influencing positively the abundance of SUP05-5 and -6 were shown to be depth, nutrients and Cd, whereas SUP05-1-4 was impacted positively mostly by DOC, H_2_S, O_2_, and DFe (Fig. S10).

#### GTDB phylogenetic analysis

**Fig. 3:**
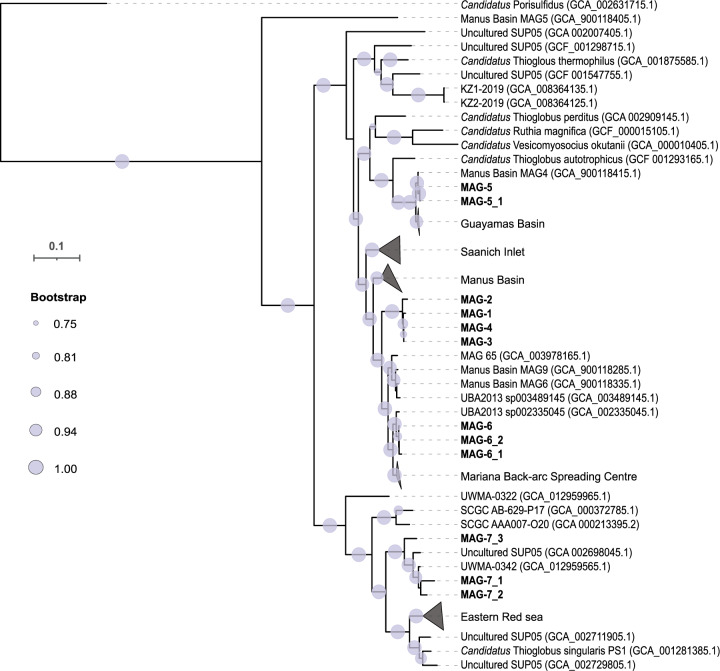
Phylogenetic analysis of SUP05 genomes. The phylogenetic tree is based on an alignment of 120 bacterial marker genes from SUP05 MAGs included in GTDB [59], 8 SUP05 MAGs obtained from the Manus Basin (PRJEB15554; ref. [58]) and 12 MAGs retrieved in this study. The tree was calculated using GTDB-Tk (https://github.com/Ecogenomics/GtdbTk). *Candidatus* Porisulfidus was used as an outgroup. Closely-related MAGs are clustered and the sample location is used as name for these clusters.

Phylogenetic analysis, based on single copy marker genes, with additional SUP05 MAGs retrieved from a previous hydrothermal study [58] and available genomes from the GTDB database [59], revealed a branching of SUP05-5 together with CTA. SUP05-1-4 was placed on a branch distant to any *Candidatus* genome (Fig. 3), hence, conflicting with 16S rRNA gene based phylogeny (Fig S1). However, in this genus, a stable branching could not be resolved either for the genome, or the 16S rRNA gene based tree, leading to generally low bootstrap support.

#### Average nucleotide identity comparison

According to ANI values, SUP05-1-4 belong to a single, not previously described species. SUP05-5 shares 95% ANI value with MAG UWMA-0078 (GCA_012964705.1) [60] and SUP05-6 belongs to the same species (96.2% ANI) as MAG UBA2013 (GCA_002335045.1) [61] (Table S5).

MAG-7_1, -7_2 and -7_3 were affiliated with CPS (Fig. 3) and were not significantly impacted by any of the recorded chemical parameters (Fig S10). They seemed to be characterized by a constantly low abundance across all samples (Table S4). Nevertheless, they seemed to be 10-fold more abundant at BrV than McV.

#### Metagenome-assembled genomes’ metabolic capabilities

**Fig. 4:**
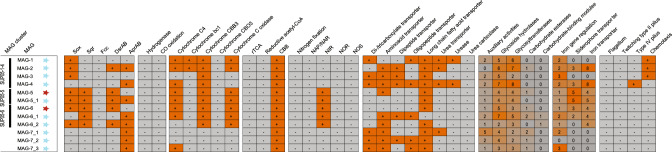
Metabolic potential of SUP05-related MAGs. Bacterial genomes were manually screened for genes for: sulfur metabolism (Sox sulfur-oxidizing enzyme, Sqr sulfide:quinone oxidoreductase, Fcc flavocytochrome *c*, Dsr dissimilatory sulfite reductase, Apr dissimilatory adenylylsulfate reductase), hydrogen oxidation and carbon monoxide oxidation, carbon fixation pathways (rTCA reverse tricarboxylic acid cycle, reductive acetyl-CoA, CBB Calvin Benson Bassham cycle), nitrogen fixation and nitrogen reduction (NAP nitrate reductase NIR nitrite reductase, NOR nitric oxide reductase, NOS nitrous oxide reductase), transporters, carbohydrate-active enzymes and lastly motility genes. MAGs with more than 95% ANI between each other are clustered together: SUP05-1-4 (MAG-1 to -4); SUP05-5 (MAG-5 and MAG 5_1); SUP05-6 (MAG-6, MAG-6_1 and MAG-6_2). MAGs retrieved from single assemblies are marked with a blue star and those resulting from a cross-assembly with a red star. The presence of genes is marked with a plus in an orange table cell, whereas the absence is marked with a minus in a gray cell. The presence of the carbohydrate-active enzymes and iron-related genes is given as a heatmap based on the number of these genes.

We analyzed in detail the metabolic capabilities of three SUP05 clusters (SUP05-1-4, SUP05-5 and SUP05-6) and MAG_7-1 to MAG_7-3 (Fig. 4). Three clusters of MAGs contained genes for the sulfur oxidation complex (SOX), such as *sox*YZ, *sox*AX and *sox*B. Genes for the reverse acting dissimilatory sulfite reductase (rDSR; *dsr*MNKJOP) were also present in those SUP05 clusters, but not detected in MAG-7_1 to -7_3. Nitrite reductase genes were encoded in SUP05-5 and SUP05-6. Furthermore, all SUP05 MAGs harbor genes encoding the RubisCO enzyme, a marker gene for the Calvin Benson Bassham cycle. SUP05-1-4 was the only cluster in which methyl-accepting chemotaxis genes could be detected. All MAGs harbored genes for amino acid and long-chain fatty acid transportation. However, SUP05-1-4 and MAG-7_1, -7_2 and -7_3 harbored the most diverse transport genes, including di-tricarboxylate, dipeptide and oligopeptide transporters. All SUP05-related MAGs contained GH23, GH73 and GH103 genes, known to degrade peptidoglycan [62, 63]. SUP05 clusters contained genes for iron transport, siderophore transport and iron gene regulation [64, 65]. Lastly, all SUP05-related MAGs had inconclusive viral genes, belonging to Category 3 (possible prediction) [66].

### Metatranscriptome analysis

In order to elucidate the set of expressed metabolic pathways, four metatranscriptomes were sequenced: two samples from McV (04CTD_b6 and 10CTD_b4) and two from BrV-NWC (54CTD_b8 and 54CTD_b12). According to 16S rRNA gene expression, SUP05 is the most active bacterial clade in the plume samples, composing 72% of the total bacterial transcripts in 10CTD_b4, 56% in 04CTD_b6, 80% in 54CTD_b8 and 60% in 54CTD_b12. Among the most expressed genes were house-keeping genes like polymerases and ribosomal proteins, and the genes involved in chemolithoautotrophy such as: sulfur oxidation genes (SOX) and RubisCO (Fig. S11).

**Fig. 5:**
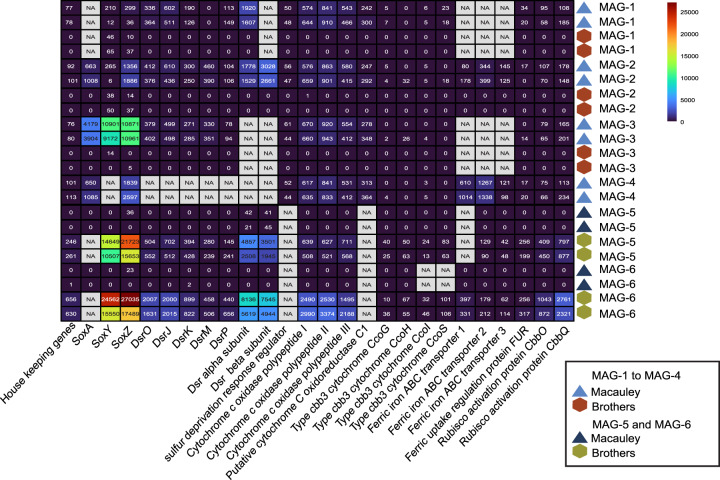
Gene expression in SUP05-related MAGs. Metatranscriptomes of Macauley and Brothers were mapped to SUP05 MAGs (SUP05-1-4, SUP05-5 and SUP05-6) using BBMap [33] with a 97% minimum identity. Transcripts were normalized to the length of the gene and the total number of reads in the metatranscriptomes (TPM). Each number represents the TPM of the gene. NA indicates that the gene was not present. Heatmap was done with pheatmap [49] in R using colorblind-friendly colors (turbo color scale) of the viridis package [50].

Moreover, the total mRNA reads were mapped unambiguously against each SUP05 MAG to investigate their expression profile. Transcripts of 04CTD_b6 and 10CTD_b4 mapped in a higher number to genes of MAG-1 to -4, reaching up to 1.7 % of total mRNA (Table S4). Whereas, 54CTD_b8 and 54CTD_b12 transcripts recruited on MAG-5 and MAG-6 genes reached up to 3.3%. As high numbers of cytochrome genes were identified in the McV metagenomes, the expression of these genes in SUP05 MAGs was inspected. For MAG-1 to -4, genes for cytochrome polypeptide I-III were highly expressed in relation to house-keeping genes (Fig. 5). According to house-keeping genes’ expression, MAG-1 to MAG-4 were not active in the BrV plumes. Simultaneously, MAG-5 and MAG-6 were not active in McV plume, but similarly active in both BrV plume samples. The expression ratio between cytochrome and house-keeping genes in MAG-1 to MAG-4 was higher compared to MAG-5 and MAG-6 (Fig. S12). SoxZ expression ratio in MAG-1 to MAG-4 was lower than in MAG-5 and MAG-6, indicating the pertinence of cytochrome genes in the McV plume.

### Global distribution of proposed new *Candidatus* Thioglobus species

**Fig. 6:**
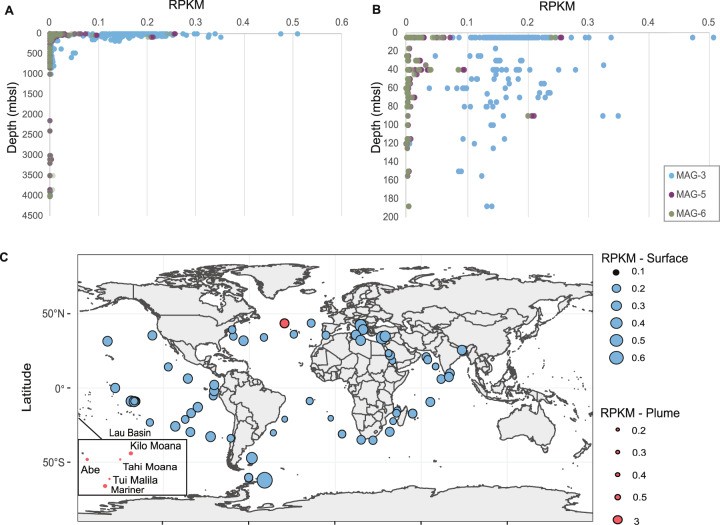
Global abundance of SUP05 MAG-3, MAG-5, MAG-6. **A** Abundance in the surface, DCM and mesopelagic metagenomes of TARA Ocean (PRJEB1787), and deep-sea metagenomes of Malaspina 2010 [67]. **B** Higher resolution of the SUP05 abundance in the first 200 meters. **C** Global distribution of MAG-3 in the surface layer and six plume metagenomes of Lau Basin (>1900 mbsl; Tai Malila, Tahi Moana, Mariner, Kilo Moana, Abe; ref. [56]) and Mid Atlantic ridge (Woody Crack—828 mbsl; ref. [43]). Reads were mapped to the SUP05 clusters using BBMap [33] with 99% minimum identity. Abundance was calculated as RPKM. MAG-3 was chosen as a representative for MAG-1-4, containing the least contamination of all four MAGs.

The global distribution of SUP05 MAGs revealed a widespread occurrence of MAG-1-4 in the surface of the open ocean (up to 0.6 RPKM) and deep chlorophyll maximum (DCM; up to 0.5 RPKM) with increased abundance values in the coastal area (Figs. 6, S13 and S14). MAG-1 to 4 showed the highest read abundance (2.6 RPKM) in the Woody Crack mesopelagic buoyant plume sampled at 828 m depth (Figs. 6 and S13). Conversely, MAG-5 and 6 had 100-fold less reads mapped to surface and DCM metagenomes (Figs. 6, S13 and S14). Nevertheless, MAG-6 prevailed in the bathypelagic plumes of Lau Basin (10.6 RPKM), whereas MAG-5 had its highest abundance in Mariner Lau Basin (8.4 RPKM) (Fig. S13). In contrast, read recruitment of the mesopelagic TARA Oceans metagenomes, and TARA Oceans metagenomes from OMZ resulted in less read recruitment for MAG-1-4 (up to 0.18 RPKM) and almost none for MAG-5 and 6 (<0.001 RPKM). Recruitment of bathypelagic Malaspina 2010 [67] metagenomes revealed only a few mapped reads for all MAGs (< 0.006 RPKM), indicating an absence from the deep-sea (Fig. 6 and Table S6).

## Discussion

Sulfur-rich plumes act as oases for chemolithoautotrophic SUP05 [56, 58, 68]. In order to better understand their diversity and ecology, we investigated the microbial communities of three plumes expelled by two submarine volcanoes with a multidisciplinary approach. Thereby, we were able to characterize and describe three new, yet uncultivated SUP05 species, *Candidatus* Thioglobus vadi (corresponding to SUP05-1-4), *Candidatus* Thioglobus vulcanius (corresponding to SUP05-5) and *Candidatus* Thioglobus plumae (corresponding to SUP05-6; Table S7). Each of these three species dominated a different plume which suggests that they partition into different environmental niches.

### SUP05 niche differentiation

As the sulfur-oxidizing SUP05 were the prominent primary producers in McV and BrV, we compared the SUP05 populations of three plumes. This comparison revealed a distinct presence of SUP05 species in bathy- and mesopelagic plumes.

The BrV was composed of two bathypelagic plumes, which were dominated by different SUP05 clusters, SUP05-5 in the BrV-cone (~1300 mbsl) and SUP05-6 in the BrV-NWC (~1600 mbsl), suggesting a niche differentiation between the two plumes. Since these MAGs fulfill the standards given in Konstantinidis et al. [69] and Murray et al. [70] for a genome-based taxonomy, we propose SUP05-5 as *Candidatus* Thioglobus vulcanius and SUP05-6 as *Candidatus* Thioglobus plumae (Table S7). Both species were more abundant in bathypelagic plumes (Abe, Mariner, Tahi Moana, Kilo Moana and Tui Manilla) compared to SUP05-1-4 but exhibited extremely low abundance in other analyzed locations (DCM, surface and deep open ocean), establishing their niche in the bathypelagic sulfur-rich plumes. Regarding the factors that drive the niche differentiation between these two species in BrV-cone and BrV-NWC, *Ca*. T. vulcanius seemed to be better equipped to endure the toxicity of heavy-metals present in the plume (Fig. S15). However, different properties such as substrate affinity and sulfide toxicity still need to be investigated [23].

**Fig. 7:**
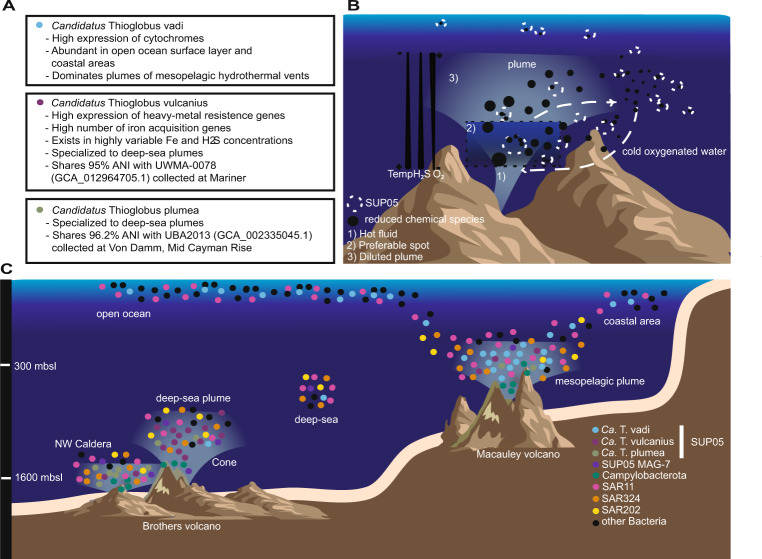
Overview of features of newly described SUP05 species and hypothesis on their distribution. **A** SUP05 clade unique features found in MAGs: *Candidatus* Thioglobus vadi, *Candidatus* Thioglobus vulcanius and *Candidatus* Thioglobus plumae, dominating different plumes. **B** Suggested schematic distribution of the SUP05 population in the cold oxygenated background water as it mixes with the hot reduced fluid and subsequently is diluted with the plume. The SUP05 population is represented with white dashed circles and the chemical reduced species are depicted in black circles. The size of the circles refers to the concentration of reduced species and the abundance of the SUP05 clade. **C** Sketch depiction of the communities in three bathy- and mesopelagic plumes. Global patterns of the SUP05 species are based on their reads per kilobase per million mapped reads (RPKM) in the surface and deep chlorophyll maximum (DCM) TARA Ocean metagenomes. Epsilonprotebacteria have recently been reclassified to Campylobacterota (phyl. nov.) [85, 86].

Secondly, SUP05-1-4 represented the dominant SUP05 species in the mesopelagic plume of McV (~300 mbsl), and were present at higher abundances in the open ocean and specifically the coastal areas. In line with our observation in McV, SUP05-1-4 also prevail in a mesopelagic buoyant plume sampled at Woody Crack in a depth of 828 m. According to ANI values, SUP05-1-4 MAGs are evidently strains of the same species. Although the completeness of these MAGs does not exceed 85%, likely reflecting the challenges arising from high strain heterogeneity, MAG-1 to 4 fulfill the standards given in Konstantinidis et al. [69] and Murray et al. [70] to be characterized as a new *Candidatus* species. Here, we propose the name *Candidatus* Thioglobus vadi, meaning bacterium “of a shallow place”. Statistical analysis suggests that this species prefers oxygenated sites with lower sulfide (highest abundance in 10CTD_b4–202.3 µM H_2_S) and higher iron concentration (10CTD_b4–22.2 nM; Fig. 2). Global distribution of *Ca*. T. vadi indicates that also depth may have a niche determining effect (Figs. 7A and S13).

SUP05 MAG-7_1, 7_2 and 7_3 affiliated to *Ca*. Pseudothioglobus singularis, a representative of the recently classified *Pseudothioglobus* genus [23]. They were characterized by high expression of oligopeptide, branched-chain amino acid and nucleoside transporters (Fig. S11c), and a missing sulfur-oxidizing (SOX) pathway. Growth experiments done by Spietz et al. [20], using the closely-related species CPS PS1, reported that carbon fixation is not critical for their growth and suggested the capacity for heterotrophy. Nevertheless, due to the low completeness of the MAGs, it is challenging to identify the lifestyle of these closely-related species.

In conclusion, niche differentiation of SUP05 within hydrothermal plumes, seems to predominantly affect chemoautotrophic subclades, whereas supposedly heterotrophic SUP05, like MAG-7_1 to MAG-7_3 are more omnipresent (Figs. 2 and 7C).

### Considering the role of iron

It was previously shown that SUP05 bacteria use energy gained from the oxidation of reduced sulfur species to fuel dark carbon fixation [71]. Particularly, the SUP05 clade is distinguished by the formation of sulfur globules and the oxidation of sulfur via the reverse dissimilatory sulfate reduction pathway (rDSR) [72]. The ability to hoard sulfur is an advantageous trait that could support a cosmopolitan and opportunistic lifestyle. In order to understand the potential preference of *Ca*. T. vadi for iron-rich niches, we conducted an in-depth investigation of its genetic potential and expression profiles.

Similar to *Ca*. T. autotrophicus, our data support the oxidation of reduced sulfur compounds with oxygen as electron acceptor. Typically, genes coding for proteins involved in sulfur oxidation are the most expressed in hydrothermal vent plumes [56] due to the thermodynamic favourability of oxidizing sulfur compounds. The total dissimilatory energy available is eight times greater for sulfide oxidation. Indeed, in *Ca*. T. vadi, sulfur oxidation related genes were among the most expressed. Also, the high mRNA expression level of cytochrome genes was noteworthy. This expression coincided with an exceptionally high number of cytochrome genes in the McV metagenomes compared to those from background seawater and other sites. Since cytochromes participate in energy conversion processes in the respiratory chain and during iron oxidation, the high expression of them in *Ca*. T. vadi could either mean that these cells were highly active, or that they were engaged in iron oxidation [64]. The first explanation is not supported by a relatively low expression of *Ca*. T. vadi house-keeping genes (Fig. S4a). Iron oxidation by *Ca*. T. vadi is also hard to verify, yet the following two considerations might make it at least plausible. Firstly, although under neutral pH, the spontaneous chemical oxidation of iron outcompetes the biological oxidation [73], in situ measurements indicate that the excess sulfide keeps Fe reduced due to a catalytic cycle. Slowly oxidizing nanoparticulate pyrite might therefore likely persist in the plume [74, 75]. Secondly, the exceptionally high expression of cytochrome oxidases genes might be linked to iron oxidation. However, the diversity of iron oxidation (FeOx) pathways and the divergence of the genes involved in iron oxidation [64, 76, 77] pose major challenges when assessing MAGs [78].

Nevertheless, we compared the SUP05 MAGs to the basic model of neutrophilic iron oxidation in Zetaproteobacteria [79]. SUP05 MAGs possess genes encoding for modules of the FeOx model, including cbb3 cytochrome *c* oxidase, *bc1* cytochrome *c* oxidase, NADH dehydrogenase and ATP synthase. Although, cytochromes reported to be involved in FeOx extracellular electron exchange such as Cyc2, Pio or MtrCAB [73] were missing in *Ca*. T. vadi MAGs, other cytochromes with multiple heme-binding motifs (-CXXCH-) [65] were expressed. Here, the function of porin-cytochromes for electron transport could be substituted by multiheme cytochromes with Fe, potentially in a pyrite form, being oxidized by a different cellular mechanism other than the one known from neutrophilic iron-oxidizing bacteria [80]. Therefore, in our case, the iron oxidation hypothesis on *Ca*. T. vadi was supported by: (1) a high number of cytochrome genes being expressed; (2) cytochromes containing heme-binding motifs, which could substitute known modules in the neutrophilic FeOx pathway; (3) positive influence of DFe concentration on *Ca*. T. vadi abundance. We conclude that, although, there are good hints for iron oxidation by *Ca*. T. vadi, these are not yet conclusive.

### Plume ecology

The ability of SUP05 to constitute up to 50% of the microbial community in an ephemeral plume remains enigmatic considering their immotility. Since SUP05 are absent in the early stages of the plume, due to suboptimal conditions, Sheik et al. [68] and Lesniewski et al. [81] suggested that vent-adapted microorganisms (SUP05) are entrained from surrounding water. This hypothesis is in line with our findings (Fig. 6) and other studies which have shown the influence of the background microbial community on the plume [4, 82]. As the background seawater contains a low number of SUP05 cells (5.94 × 10^2^ cell/ml—04CTD_b4; Fig. S4), only a low number of cells entrains the plume. In the plume, SUP05 reaches up to 1.56 × 10^4^ cell/ml (04CTD_b6), suggesting that they can react swiftly to reduced chemical species and are capable of rapid growth in their preferable spot in the hydrothermal plume (Fig. 7B). We observed that the absolute cell concentration of SUP05 in plumes was independent of depth (BrV—054CTD_b8: 2.68 × 10^4^ cell/ml; McV—10CTD_b4: 3.49 × 10^4^ cells/ml), although, due to the low number of cells in the deep-sea, the 16S rRNA analysis gives the impression that the bathypelagic plumes have a higher abundance of SUP05 (Fig. 2). On the contrary, the SUP05 “preferable spot” is an ephemeral site, which is characterized by low temperature, high oxygen concentration and low concentration of reduced sulfur species [58]. Shah et al. [71] showed that concentrations as low as 10 nM of reduced sulfur could support sulfur oxidation in oxygenated seawater. Therefore, owing to their ability to use miniscule amounts of reduced chemical species and to store sulfur [71, 83], SUP05 persevere in the background water (Fig. 6) as the cold water mixes with the hot reduced fluid (Fig. 7). Nevertheless, the dilution of the plume and the depletion of reduced chemical species, is followed by a reduction in SUP05 abundance. This could be observed in the 54CTD samples, where the vertical profile of SUP05 abundance closely resembles the vertical profile of the plume indicators (Table S8).

As the plume gets diluted, background seawater could feedback into the plume and re-introduce microorganisms [68], especially since the age of the plume is shown to be up to ~30 days [84]. Due to this cycle of entrainment and dilution of the vent-adapted microorganisms such as SUP05, the plume acts as growth chambers for SUP05, from which they are released into the surrounding water and could re-inoculate plumes.

## Conclusion

By applying several complimentary culture-independent techniques, supported by an extensive set of geochemical measurements, we could shed light into the ecology of three novel candidate species of the clade SUP05. As expected, reduced chemical compounds present in the plume seem to have a significant influence on niche differentiation. Water depth seems to be another important factor. We show that the bathypelagic plumes are dominated by two different, yet uncultivated SUP05 species, *Candidatus* Thioglobus vulcanius, and *Candidatus* Thioglobus plumae, whereas the mesopelagic plume is dominated by the also yet uncultivated species *Candidatus* Thioglobus vadi. Knowing the physico-chemical characteristics of the environment, it is possible to predict the dominant SUP05 species in plumes of different hydrothermal systems, and vice versa certain SUP05 species might be indicators for the prevailing plume characteristics.

## Supplementary information

Supplementary Information

Supplementary Figures
