# Supplementary material for: Niche differentiation of sulfur-oxidizing bacteria (SUP05) in submarine hydrothermal plumes
Source: ISME J. 2022 Jan 26;16(6):1479–90. doi: 10.1038/s41396-022-01195-x (PMC9123188; doi:10.1038/s41396-022-01195-x)
Supplement: Supplementary file 1 — Supplementary Information [file 41396_2022_1195_MOESM1_ESM.pdf]

Supplementary Information

Niche differentiation of sulfur-oxidizing bacteria (SUP05) in submarine hydrothermal plumes

Bledina Dede, Christian T. Hansen, Rene Neuholz, Bernhard Schnetger, Charlotte Kleint, Sharon Walker, Wolfgang Bach, Rudolf Amann, Anke Meyerdierks

Supplementary Materials and Methods

Sample collection

Monitoring the primary plume tracers of turbidity and ORP in real-time directed the discrete sampling of both hydrothermal plume and non-plume affected water.

Turbidity anomalies are reported as dimensionless Nephelometric Turbidity Units [1] above the regional background (ΔNTU). The ORP sensor detects reduced hydrothermal chemical species (e.g., Fe^{2+}, HS^-, H_2) that oxidize rapidly in dispersing neutrally-buoyant hydrothermal plumes [2,3]. ORP anomalies are identified by a negative change in potential (E, mV) and are expressed as either the time derivative (dE/dt, mV) that shows a sustained decreasing trend greater than the ambient drift over time, or as the magnitude and duration of the overall drop in value for any given signal (ΔE, mV).

After retrieval of the CTD rosette on board, water samples were immediately processed for further analyses: A small volume of each sample (2 l) was filtered through 0.22 pore size polycarbonate (PC) membrane filters (4.7 mm diameter, Millipore, Darmstadt, Germany). In addition, from selected samples, large volumes of 10-30 litre were filtered through 14.2 cm diameter PC filters (0.22 µm pore size), with the exception of 54CTD_b18 which was filtered through a polyethersulfone (PES; Millipore, Darmstadt, Germany) filter (0.22 µm pore size). All filters were sliced and preserved for different downstream analyses: Subsamples for DNA and RNA extraction were either directly stored at -80°C, or preserved with RNAlater prior to freezing. For fluorescence *in situ* hybridization (FISH), samples were fixed with formaldehyde (1% final concentration) for 10-16 h at 4°C. Subsequently, cells were collected by filtration on 0.22 µm pore size PC membrane filters, dried and frozen until use.

DOC and SPE-DOC, and nutrients were measured on samples, collected at the same time and depth in parallel Niskin bottles. For example, Niskin bottles 04CTD_b1 and b2 were closed at the same time. Subsequently, the 04CTD_b1 sample was used for geochemical analyses, whereas 04CTD_b2 was processed for culture-independent molecular techniques in this study.

16S rRNA gene amplification

DNA was extracted either from 1⁄4 − 1⁄3 of a 4.7 cm PC filter piece or a 1-2 cm^2 piece of a 14.2 cm PC membrane filter using the PowerSoil DNA Isolation Kit (MoBio, CA, USA) according to the manufacturer's instructions. The V3-V4 region of the 16S rRNA gene was amplified using the primer combination Bakt_341F and Bakt_805R [4] using barcoded primers [5]. Phusion-PCR was conducted as follows: Each 25 µl PCR reaction mix was composed of 0.75-1.5 µl of template DNA, in one fold concentrated Phusion DNA polymerase HF buffer, containing 0.2-0.4 µM of each primer, 200 µM dNTP, 5% (v/v) dimethyl-sulfoxide, 0.02 U/µl Phusion DNA polymerase (Thermo Fisher Scientific, Waltham, MA USA) and additional 1.5 mM $MgCl_2$. Cycling conditions were as follows: 30 s denaturation at 98°C followed by 30-35 cycles of 10 s denaturation at 98°C, 30 s annealing at 55°C and 15 s elongation at 72°C, and a 10 min final elongation step at 72°C. Only 16S rRNA genes of 10CTD samples were amplified using TaKaRa Ex Taq DNA polymerase (Takara, Kusatsu, Japan) according to the following protocol: 1 µl DNA template in a 25 µl PCR reaction containing 0.3 mg/ml bovine serum albumin, 200 µM of each dNTP, 1 µM of each primer, 0.05 U/µl TaKaRa Ex Taq DNA polymerase (Takara) in 1-fold concentrated Ex Taq buffer. The PCR cycling conditions were as follows: 3 min denaturation at 96°C followed by 30 cycles of 1 min denaturation at 96°C, 1 min annealing at 55°C and 2 min elongation at 72°C, and a 10 min final elongation step at 72°C. PCR reactions were checked using an E-Gel iBase and E-Gel Safe Imager system (Thermo

Fisher Scientific) and amplicons were purified using AMPure XP (Beckmann Coulter, Brea, CA, USA). PCR product concentrations were determined with a Qubit 3.0 fluorometer and the Qubit dsDNA HS Assay Kit (Invitrogen). Equimolar amounts of PCR product were pooled for amplicon sequencing on an Ion Torrent Personal Genome Machine (PGM) System (Thermo Fisher Scientific) using Ion PGM Hi-Q chemistry. Part of DNA extractions, PCR reactions and amplicon sequencing runs were already conducted on-board of cruise SO253 and parts were sequenced in the home laboratory.

Metagenome sequencing and analysis

The protocol "Ovation Ultralow System v2 1-16" (NuGEN, Redwood City, CA, US) was followed for end-repair, ligation and ligation purification. Fragments which contained the adaptors were amplified via 14 PCR cycles. The resulting libraries had an insert size of 489-614 bp and a final DNA concentration of 10.6-30.6 ng/µl. The fragments were sequenced (2x250 bp length) on a HiSeq 2500 sequencer (Illumina, San Diego, CA, USA) at the Max Planck Genome Centre (Cologne, Germany).

Additionally, a community-wide functional analysis between McV (five metagenomes), BrV (three metagenomes), Mariner (DOE JGI-IMG/MER-Taxon object ID 3300001678), Kilo Moana (DOE JGI- IMG/MER-Taxon object ID 3300001680) plume (Eastern Lau Spreading Centre, ELSC; ref. 6), Woody Crack buoyant plume (SAMEA3597868) (Menez Gwen, Mid-Atlantic Ridge; ref. 7) and South Pacific Ocean background water at 201 m (SAMN07136823) and 1023 m (SAMN07136798) [8] depth was conducted, in order to compare the abundance of functional genes between these plumes. Genes were normalized using the ribosomal gene *rpo*B. Mariner and Kilo Moana plume [6] as well as Woody Crack buoyant plume [7] metagenomes were chosen due to the chemical similarity of the vent fluids to those emitted at BrV and McV. Functional analyses were done by gene comparison against the Uniref100 Release 2019_04

database using DIAMOND v0.8.24.86 (e-15) [9] and visualization of the results using MEGAN v6.6.0 [10]. MEGAN was further used for a neighbor joining analysis of the dissimilarities and a biplot PCoA. Relative numbers of sox and cytochrome genes were compared between all metagenomes.

16S rRNA sequences were sorted across all metagenomes with SortMeRNA v2.0 [11]. These sequences were clustered and taxonomically classified with SilvaNGS [12] using SILVA SSU132 taxonomy. Moreover, phyloFlash v3.3b1 [13] reconstructed 16S rRNA fragments between 900 to 1500 bp.

All MAGs were visualized and manually refined using anvi'o v4 and v6 [14]. Completeness and contamination of the MAGs was analyzed by CheckM [15]. A genome-based maximum likelihood phylogenetic tree of SUP05 MAGs and publicly available genomes [16] was calculated using an alignment of 120 bacterial marker genes by HMMER [17] in GTDB-Tk v1.3.0 [18]. The tree was visualized using ITOL [19]. Average nucleotide identity of retrieved MAGs was calculated by JSpeciesWS [20]. MAGs were automatically annotated with RAST [21] and Prokka [22]. Metabolic capabilities of MAGs were manually checked and genes in agreement between pipelines were depicted as present. Carbohydrate-active enzymes were checked by blasting against the Carbohydrate Active Enzymes Database (default parameters; http://www.cazy.org/) [23]. Iron-related genes were checked using the program FeGenie [24]. Viral genes were mined using VirSorter v2 [25].

Furthermore, MAGs retrieved in this study were mapped to the surface, deep-chlorophyll maximum and mesopelagic metagenomes collected during the TARA Ocean expedition 2013 (PRJEB1787), bathypelagic metagenomes of the Malaspina 2010 dataset [26] and plume metagenomes from Lau Basin (Tui Malila, Tahi Moana, Kilo Moana, Mariner and Abe; ref. 6)

and Mid-Atlantic Ridge (Woody Crack; ref. 7), using using BBMap [27] with 0.99 minimum identity.

Metatranscriptome sequencing

Total RNA was extracted from 4-15 cm^2 membrane filter pieces according to the following protocol: Samples were thawn on ice and RNAlater was removed carefully (exception: 54CTD_B12 was not preserved in RNAlater, but directly frozen and stored at -80°C after filtering). Filter pieces were transferred to a 5 ml polypropylene (PP) tube. 1.8 ml ROTI Aqua-P/C/I (Carl Roth, Karlsruhe, Germany), 1.8 ml extraction buffer [120 mM sodium phosphate, pH 8, 1% acid washed polyvinylpolypyrrolidone (PVPP)], 260 µl 10% SDS and the content of 1 tube Lysing matrix E (MP Biomedicals, Irvine, CA, USA) were added. Tubes were shaken on a Vortex Genie (Scientific Industries, Bohemia, NY, USA) at maximum speed for 10 min, centrifuged at 5242xg for 10 min at room temperature, and the aqueous phase was transferred to a fresh tube. The organic phase was re-extracted with 500 µl 100 mM sodium phosphate buffer (pH 8), including an additional 10 min vortex step, 10 min incubation at 60°C, and a further 3 min vortex step. The aqueous phases of both extractions were combined, an equal volume of ROTI Aqua-P/C/I (Carl Roth) added, and the solution was mixed and centrifuged as before. The aqueous phase was again transferred to a fresh PP tube and nucleic acids were precipitated by adding 0.1 volume 3 M sodium acetate and 0.7 volumes isopropanol, mixing, incubating for 1 h on ice and 30 min centrifugation at 21000xg and 4°C. The pellet was washed with ice cold 75% ethanol, and the nucleic acids were resuspended in 50 µl RNase free water. Furthermore, DNA was removed by DNase treatment (Turbo DNase, Thermo Fisher Scientific) and total RNA was purified by columns (RNA clean and concentrator, Zymo Research, Irvine, CA, USA). Capillary electrophoresis (Picochip, Agilent Bioanalyser; Agilent, Santa Clara, CA, USA) was used for quality assessment. An Illumina-compatible library

was generated with the NEBNext Ultra II Directional RNA Library Prep kit (NEB, Ipswich, MA, USA) followed by sequencing (2x250 bp) on a HiSeq 2500 system (Illumina) at the Max Planck Genome Centre (Cologne, Germany). We received more than 24 million reads per sample (Table S3).

Phylogenetic reconstruction

SUP05 16S rRNA sequences were aligned to a curated SILVA SSU132 NR99 database (alignment quality >85, pintail >50) using SINA [28]. Phylogenetic trees were constructed based on almost full length SUP05 16S rRNA genes reconstructed with phyloFlash [13] (length > 900 bp) and 60 related published sequences (quality >98) in ARB [29]. Several treeing algorithms such as: Neighbour-joining [29], PhyML [30] and RAxML [31] and different position conservation filters (no filter, 30%, 50%) were applied. Shorter 16S rRNA amplicon sequences and 16S rRNA gene sequences retrieved from MAGs were added to the trees using maximum parsimony, without allowing changes of the overall tree topology. Sequences were exported from the tree and used to calculate the abundance of the SUP05 subgroups: CTA, CTT and CPS. Species representative of each group are CTA [32], CTT [33] and CPS [34].

CARD-FISH

CARD-FISH analysis was conducted according to Pernthaler et al. [35]. Cell permeabilization was conducted at 37°C for 60 min with 10 mg/ml lysozyme. Additionally, archaeal cell walls were permeabilized with 15 µg/ml proteinase K for 3 minutes at room temperature. Endogenous peroxidases were inactivated with 0.1 M HCl for 10 min. Counterstaining was done with 1 µg/ml 4',6- diamidino-2-phenylindole (DAPI).

Probes used in this study: EUBI-III mix [36,37], NON338 [38], Arch915 [39], GAM42a with competitor BET42a [40] and SUP05_1241 [7]. SUP05_1241 probe was checked on 16S rRNA

genes reconstructed from metagenomic reads. Other Gammaproteobacteria cells were determined using the Gam42a probe, including competitor. This probe does not target SUP05 cells, confirmed by a probe match against reconstructed 23S rRNA sequences. Filters were counted using a Zeiss Image 2D or Nikon Eclipse 50i microscope with a 1000x magnification. Depending on the abundance of the cells and the intensity of the signal 5 rows of a grid or a whole grid (up to 1000 cells) were counted in 8- 10 different fields of view.

Thermodynamic calculations and Statistical analysis

Fe concentration in plumes was used to compute ΔrG, whereas for H_2S contents of the plume waters, it was assumed that the Fe:H_2S ratio in the plumes is identical to the Fe:H_2S ratio in the vent fluids. The following concentration values for H_2S and Fe in BrV and McV vent fluids were used: McV: 1.7 mM Fe, 15.5 mM H_2S; BrV-NWC: 8.6 mM Fe, 3.1 mM H_2S; BrV-Cone: 0.15 mM Fe, 5.0 mM H_2S [41]. An O_2(aq) concentration of 0.15 mM was assumed for all plume water samples. The concentration of the limiting compound in the catabolic reaction was multiplied by the Gibb's free energy to determine the energy available per mol reaction in one kg of plume water. These values were divided by the number of electrons transferred in sulfide oxidation (8) and iron oxidation (1).

Statistical analyses were performed in R. PerMANOVA [42] was used to test the redundancy analysis and to analyse the impact of environmental conditions. Linear regression using the package "stats" v3.6.2 [43] was conducted to model the relationship between specific clades and chemical parameters. Linear model assumption was validated using the gvlma v1.0.0.3 package [44]. Similarity percentages breakdown analysis was performed to discriminate species contributing to the dissimilarity using "Simper" function [45].

Chemical measurements

DOC and TDN

Dissolved organic carbon (DOC) and total dissolved nitrogen (TDN) were quantified on triplicate 10 mL water samples by high temperature catalytic combustion (Shimadzu TOC-VCPH) in the Marine Chemistry lab at the ICBM of the University of Oldenburg following on-board filtration (0.2 µm, GHP Aerodisc), acidification (pH2, 0.01 M HCl) and dark, refrigerated transport. Additional water volumes were solid-phase extracted (SPE) following the procedure described in Dittmar et al. [46] to acquire a methanolic extract containing a subset of DOC (SPE-DOC) which is less prone to contamination and can be further characterized on a molecular level. SPE-DOC was measured as bulk DOC following evaporation of an extract aliquot and re-dissolution in 0.01 M HCL as described in Hansen et al. [47]. Accuracy (< 2% deviation of expected reference value), precision (RSD% < 4%) and LOD (1 µmol L-1) of the analysis were monitored via repeated measurements of ultrapure water and a deep-sea reference material (Hansell, Florida Strait, 700 m water depth, batch No. 9, 2009, 44 µmol DOC L−).

DFe

Dissolved Fe concentrations were determined after pre-concentration prior to analysis with high- resolution sector field inductively coupled plasma-mass spectrometry (HR-SF-ICP-MS, Thermo Fisher Element 2, Walsham, MA, USA) at the Royal Netherlands Institute for Sea Research (NIOZ). Detailed information on sample collection, processing and analysis can be found in Neuholz et al. [48].

Macronutrient analysis

From CTD casts an aliquot of 25 mL was immediately filtered through 0.45 µm surfactant-free cellulose acetate (SFCA) syringe filters into high-density polyethylene (HDPE) vails. Concentrated $HgCl_2$ solution (150 µL) was added to a final concentration of 0.4 mM $HgCl_2$ to filtered samples to stop biologic activity before storing samples cold and dark until further analysis. Within three days after collection, samples were analyzed on board for NOx (nitrate + nitrite), PO_4 using colorimetric methods on a 96-well microplate spectrophotometer (Multiscan GO, Thermo Scientific). Precision and trueness were better than 10% and checked with independent in-house reference solutions of known concentrations and matrix. NOx was analyzed as described in Schnetger and Lehners [49] with a limit of detection (LOD) of 0.32 µM and limit of quantification (LOQ) of 0.98 µM. Phosphate concentrations were analyzed using the general method of O`Dell [50] with LOD of 0.04 µM and LOQ of 0.13 µM.

Supplementary tables

Table S1. Main environmental parameters for samples used in this study. Geo-chemical parameters were measured on board using in-line temperature and pH probes and in the lab after sample collection. As sulfide was below the detection limit in the plume, the highest measured concentration of DFe and H_2S in the fluid coupled with maximum temperature were used to calculate plume sulfide concentrations. The maximal concentrations were chosen, due to our sampling strategy, which was geared towards sampling lower-T fluids, subsequently lowering the Fe and H_2S average in the vent fluid.

Sample	Source	Cast type	Plume area	ΔNTU	Longitude	Latitude	Depth [m]	Temp. [°C]	pH	O_2 [µmol/kg]	DFe [nM]	DOC [µM]	TDN [µM]	PO_4 [µM]	NOx [µM]	H_2S [µM]	SPE-DOC [µM]	CARD FISH/ Metagenome/ Metatranscriptome
**01CTD_b19**	Background	Vertical	Backg.	NA	-179.0751	-28.259	402	13.6	NA	184.2	NA	NA	NA	NA	NA	NA	NA	- / - / -
**79CTD_b6**	Background	Vertical	Backg.	NA	-178.7771	-35.2584	1499	3.8	7.8	159.9	NA	172	39	NA	NA	NA	20	- / - / -
**04CTD_b2**	Macauley	Tow-yo	Plume	0.0534	-178.44242	-30.2115	248	15.1	8	186.7	7.2	177	10	0.4	7.1	65.7	2.78	+ / - / -
**04CTD_b4**	Macauley	Tow-yo	Above	0.0021	-178.44794	-30.21316	221	15.6	8	188.3	3.8	194	10	0.4	5.9	34.3	31	+ / - / -
**04CTD_b6**	Macauley	Tow-yo	Max	0.2021	-178.44961	-30.21332	287	15	7.9	178.7	126	199	11	0.5	8.1	1148.8	19.8	+ / + / +
**04CTD_b8**	Macauley	Tow-yo	Below	0.0055	-178.45427	-30.21469	351	13	8	187.1	6	169	14	0.7	12	54.4	26.4	+ / - / -
**04CTD_b10**	Macauley	Tow-yo	No sig.	0.0024	-178.45589	-30.2153	250	15	8	187.1	3.8	162	10	0.5	7	34.3	26.7	+ / - / -
**10CTD_b2**	Macauley	Tow-yo	Plume	0.0345	-178.44357	-30.21458	253	15.1	8	187.7	9.5	NA	NA	0.5	7	87.1	NA	+ / + / -
**10CTD_b4**	Macauley	Tow-yo	Plume	0.2777	-178.44933	-30.21293	274	14.8	7.8	183	22.2	291	11	0.6	7.4	202.3	27	+ / + / +
**10CTD_b10**	Macauley	Tow-yo	Above	0.0227	-178.45055	-30.21263	195	16.2	7.9	188.1	6.1	299	9	0.3	5.5	55.2	24	+ / + / -
**10CTD_b12**	Macauley	Tow-yo	Plume	0.0654	-178.45337	-30.21177	264	14.9	7.9	186.8	5.5	NA	NA	0.5	6.8	50	NA	+ / + / -
**10CTD_b17**	Macauley	Tow-yo	Weak	0.026	-178.45924	-30.21	274	14.9	7.9	186.8	12.8	257	17	0.9	8.5	NA	23	+ / - / -
**10CTD_b19**	Macauley	Tow-yo	No sig	0.0186	-178.46741	-30.2076	276	14.8	7.9	186.4	12.5	242	11	0.5	8.1	NA	28	+ / - / -
**49CTD_b2**	Brothers NWC	Tow-yo	Plume	0.0208	179.0898	-34.8527	1335	4.2	7.8	168.3	6	45	33	2.2	31.9	2.2	22	+ / - / -
**49CTD_b4**	Brothers NWC	Tow-yo	Plume	0.0107	179.0894	-34.863	1496	3.3	7.8	155.04	9.6	43	35	2.2	31.4	3.5	19	- / - / -
**49CTD_b6**	Brothers UP	Tow-yo	Above	0.0118	179.082	-34.87	1229	4.9	7.8	178.3	20.6	47	31	2.1	28.1	7.4	20	- / - / -
**49CTD_b8**	Brothers NWC	Tow-yo	Plume	0.1103	179.081	-34.87	1538	3.4	7.8	156	15.3	44	34	2.3	31.3	5.5	20	- / - / -
**49CTD_b10**	Brothers LC	Tow-yo	Plume	0.0042	179.075	-34.876	1308	4.5	7.8	171.1	11.7	44	33	2.2	30.8	4.2	20	- / + / -
**49CTD_b12**	Brothers LC	Tow-yo	Plume	0.0136	179.075	-34.877	1280	4.6	7.8	171.6	25.1	44	32	2.1	29.7	9	20	- / - / -
**49CTD_b16**	Brothers LC	Tow-yo	Plume	0.0226	179.07	-34.88	1304	4.3	7.6	167.6	18.8	48	33	2.1	29.6	6.8	23	- / + / -
**49CTD_b18**	Brothers NWC	Tow-yo	Weak	0.0049	179.055	-34.9	1556	3.2	8.02	153.4	1.73	59	10	0.5	5.5	0.6	24	- / - / -
**54CTD_b4**	Brothers NWC	Vertical	Plume	0.0466	179.062	-34.87	1750	3.3	7.7	153.1	15.5	42	35	2.2	35	5.6	20	+- / - / -
**54CTD_b8**	Brothers NWC	Vertical	Plume	0.1222	179.062	-34.87	1560	3.4	7.8	154.7	34.6	42	34	2.2	35	12.5	19	+ / + / +
**54CTD_b12**	Brothers NWC	Vertical	Plume	0.0475	179.062	-34.87	1440	3.5	7.8	157.8	12.7	44	35	2.2	34.7	4.6	19	+ /+ / +
**54CTD_b15**	Brothers NWC	Vertical	Between	0.0025	179.062	-34.87	1371	3.8	7.8	152.5	6.8	42	34	2.2	34.3	2.4	20	+ / - / -
**54CTD_b18**	Brothers UP	Vertical	Above	0.0124	179.063	-34.87	1229	4.6	7.8	147.7	22.2	46	33	2.08	32.3	8	21	- / - / -

Brothers NWC. = Brothers NW Caldera; Brothers LC = Brothers lower cone; Brothers UP = Brothers upper cone

Table S2. Linear regression between SUP05-related OTUs and geochemical parameters. Analyses were calculated based on a Bray-Curtis dissimilarity matrix and standardized log environmental parameters.

Location	Taxa	Variable	Estimate	*P*-value
Macauley	CTA	H_2S	-219.01	0.02
		DFe	219.3	0.02
		O_2	18.6	0.003
		Temperature	0.7	0.05
	CTT	O_2	4.5	0.09
Brothers	CTT	Depth	1.7	0.07
		TDN	-3.8	0.01
		PO_4	3.5	0.009
	CPS	pH	-0.8	0.012

Table S3. Metagenome and metatranscriptome sequencing, bulk assembly statistics of the metagenomes and clean mRNA sorted from rRNA using SortMeRNA.

	Metagenome					Metatranscriptome	
**Samples**	**Raw Reads (2x250bp)**	**Coverage**	**N50**	**Contig > 5000 bp**	**GC (%)**	**Raw Reads**	**Sorted mRNA**
**04CTD_b6**	3.27E+07	43%	1474	1153	43.38	4.43E+07	3.97E+06
**10CTD_b2**	2.93E+07	45%	1513	1160	43.69	-	-
**10CTD_b4**	2.81E+07	48%	1506	931	42.92	2.43E+07	2.10E+06
**10CTD_b10**	3.11E+07	41%	1490	1101	43.8	-	-
**10CTD_b12**	2.73E+07	43%	1495	1083	43.87	-	-
**49CTD_b10**	3.25E+07	60%	1591	1687	47.04	-	-
**49CTD_b16**	3.24E+07	72%	1628	1735	47.98	-	-
**54CTD_b8**	3.09E+07	68%	1579	1033	46.93	5.38E+07	4.30E+06
**54CTD_b12**	3.30E+07	68%	1594	1409	46.56	5.20E+07	1.00E+07

Table S4. MAG completeness, contamination and relative abundance in the metagenomic datasets. a) Relative abundance and statistics of SUP05-related MAGs in the raw read datasets of different metagenomes. MAGs with more than 99% ANI between each other are clustered together: SUP05-1-4 (MAG-1 to -4); SUP05-5 (MAG-5 and MAG 5_1); SUP05-6 (MAG-6, MAG-6_1 and MAG-6_2). The left panel shows the percentage of raw reads mapped to binned contigs with 97% identity. The middle panel shows completeness and contamination of MAGs calculated by CheckM, and the right panel shows MAG composite information. All MAGs were retrieved from single assemblies, apart from MAG-5 and MAG-6. b) Relative abundance of MAGs which affiliated taxonomically with other taxa. c) Statistics of relative abundance of SUP05 clusters in the metatranscriptomes, with minimum identity of 97% identity, calculated as read percentage and RPKM. The darker color of the heatmap signals higher RPKM numbers.

A

		Macauley					Brothers lower cone		Brothers caldera							
MAG Cluster	MAG	4CTD_b6	10CTD_b2	10CTD_b4	10CTD_b10	10CTD_b12	49CTD_b10	49CTD_b16	54CTD_b8	54CTD_b12	Completeness	Contamination	GC content	Genome size	Number of contigs	N50
SUP05-1-4	MAG-1	8.8	13.8	15.8	0.1	3.2	0.1	0.0	0.4	0.4	75.1	8.3	38%	1048098	105	23510
	MAG-2	6.1	9.5	10.6	0.1	2.2	0.2	0.2	0.3	0.4	78.2	10	39%	1193361	283	8130
	MAG-3	7.3	11.5	12.8	0.0	2.5	0.0	0.0	0.3	0.3	73.8	1.7	38%	985958	180	10251
	MAG-4	7.1	10.9	12.2	0.1	2.4	0.1	0.1	0.2	0.3	70.1	3.6	38%	1185126	157	46271
SUP05-5	MAG-5	0.0	0.1	0.1	0.0	0.0	8.3	7.2	49.9	25.0	94.1	0	38%	1131004	86	26007
	MAG-5_1	0.0	0.0	0.0	0.0	0.0	6.4	5.6	34.4	19.0	85.6	0.8	38%	988981	257	4793
SUP05-6	MAG-6	0.1	0.1	0.1	0.0	0.0	2.1	1.9	93.5	42.3	91.5	0.8	40%	1013498	82	18988
	MAG-6_1	0.0	0.0	0.0	0.0	0.0	1.4	1.4	35.2	19.1	57.0	8.8	40%	1015596	435	2512
	MAG-6_2	0.1	0.1	0.1	0.0	0.0	1.5	1.3	59.1	30.8	59.0	1.6	40%	673393	261	2902
	MAG-7_1	0.0	0.0	0.0	0.0	0.0	0.9	0.8	1.0	1.5	61.5	11	37%	985035	365	3157
	MAG-7_2	0.0	0.0	0.0	0.0	0.0	0.8	0.7	0.8	1.2	62.4	8.9	37%	1040557	464	2573
	MAG-7_3	0.1	0.1	0.1	0.0	0.1	1.0	1.0	0.8	1.4	53.0	10.8	38%	762375	328	2732

B

uncultured *Chloroflexi*	0.1	0.1	0.3	0.1	0.1	0.0	0.0	0.0	0.0	50.0	6	52%	1946800	1518	1236
uncultured *Acidimicrobiales*	1.1	0.7	1.0	0.7	0.7	0.1	0.0	0.0	0.0	66.0	7	67%	1553584	693	2496
SAR324	0.0	0.0	0.0	0.0	0.0	0.2	0.1	0.2	0.2	66.0	3	68%	2776919	551	5267
Sulfurimonas	0.0	0.0	0.0	0.0	0.0	0.5	0.4	0.5	0.5	52.0	0	35%	961335	216	4475
Erythrobacter	0.0	0.0	0.0	0.0	0.0	0.1	0.1	0.1	0.1	51.0	7	62%	1938632	527	3563

C

	4CTD_b6		10CTD_b4		54CTD_b8		54CTD_b12	
	Rel.abud. (%)	RPKM	Rel.abud. (%)	RPKM	Rel.abud. (%)	RPKM	Rel.abud. (%)	RPKM
SUP05-1-4	1.2	5.3	1.7	7.9	0.0	0.0	0.0	0.0
SUP05-5	0.0	0.0	0.0	0.0	2.1	10.0	0.3	1.5
SUP05-6	0.0	0.1	0.0	0.0	3.3	18.1	0.5	3.0

Table S5. Average nucleotide identity (ANI) of SUP05 MAGs retrieved in this study and published genomes [15,53]

	MAG-1	MAG-2	MAG- 3	MAG-4	MAG-5	MAG-5_1	MAG-6	MAG-6_1	MAG-6_2	MAG-7_1	MAG-7_2	MAG-7_3	UWMA-0078	UBA2013	CTA EF1 [T]	CPS PS1	CTT
MAG-1	*	95.18	96.31	95.88	74.38	74.54	80.32	79.92	80.72	68.78	68.78	68.71	75.18	80.06	73.96	67.85	73.42
MAG-2	94.48	*	94.57	94.54	73.74	73.81	79.44	79.47	80.1	70.44	70.81	70.77	74.67	79.28	73.36	68.94	73.05
MAG-3	96.9	96.01	*	95.82	74.34	74.46	80.65	80.35	80.81	68.71	68.96	68.81	75.54	80.19	73.83	67.55	73.45
MAG-4	96.89	96.13	96.51	*	73.42	73.6	80.19	79.69	80.7	69.6	69.6	69.46	74.4	80.07	73.16	68.26	73.2
MAG-5	74.4	73.8	73.86	73.45	*	99.47	74.52	77.43	75.04	68.29	68.31	68.57	94.83	74.25	77.3	67.47	72.14
MAG-5_1	74.61	74.08	74	73.88	99.49	*	74.64	76.13	75.02	68.55	68.76	68.8	95	74.51	74.65	68.36	74.73
MAG-6	81.14	80.53	81.19	81.07	74.41	74.67	*	97.85	99.36	68.44	68.71	68.93	77.8	96.2	77.62	67.74	72.53
MAG-6_1	80.13	79.83	80.18	79.83	76.95	75.78	96.4	*	96.98	69.9	69.9	70.26	79.38	93.82	73.95	67.74	74.12
MAG-6_2	81.61	81.03	81.72	81.58	75.24	75.14	99.52	99.03	*	68.77	68.87	68.58	78.63	96.76	74	68.36	73.64
MAG-7_1	68.9	70.39	68.49	69.56	68.33	68.48	68.3	70.06	68.8	*	95.33	93.92	68	68.25	68.06	74.56	68.14
MAG-7_2	68.67	70.77	68.8	69.42	68.63	68.81	68.54	69.91	69.03	94.54	*	93.34	68.31	68.5	68.46	74.73	68.42
MAG-7_3	68.8	70.83	68.62	69.5	68.41	68.66	68.64	70.64	68.7	93.9	93.87	*	68.3	68.52	68.42	74.32	68.33
UWMA-0078	75.37	74.72	75.41	74.67	92.5	92.73	77.9	78.98	78.33	68.01	68.21	68.08	*	77.71	77.21	67.32	72.62
UBA2013	80.84	80.44	81.04	80.86	74.25	74.39	96.2	94.98	96.7	68.38	68.68	68.55	77.94	*	73.76	67.48	73.99
CTA EF1	73.83	73.19	73.6	73.23	77.31	77.48	73.95	74.14	74.53	67.91	68.32	68.17	77.44	73.83	*	67.38	72.57
CPS PS1	67.93	69.17	67.73	68.41	67.57	67.81	67.76	68.49	68.38	74.77	74.63	74.15	67.45	67.56	67.33	*	67.57
CTT	73.74	73.33	73.92	73.44	72.34	72.51	74.16	74.08	74.68	68.08	68.05	68.26	72.61	73.92	72.74	67.66	*

Threshold according to Goris et al. [51]: Genus >70%; Species >95%
CTA: *Candidatus* Thioglobus autotrophicus; CPS: *Candidatus* Pseudothioglobus singularis; CTT: *Candidatus* Thioglobus thermophilus

Table S6. RPKM abundance of SUP05 in mesopelagic, bathypelagic and oxygen minimum zone metagenomes of TARA Ocean and Malaspina datasets. Reads were mapped to the SUP05 MAGs using BBMap [27] with 99% minimum identity. Abundance was calculated as RPKM.

Dataset	Run	Depth	latitudeN	longitudeE	Total Reads	SUP05-1	SUP05-2	SUP05-3	SUP05-4	SUP05-5	SUP05-6
TARA	ERR598944	800	43.72	-16.87	3.46E+08	0.008	0.140	0.003	0.073	0.000	0.000
TARA	ERR598947	700	-31.02	4.67	3.16E+08	0.009	0.183	0.005	0.080	0.000	0.000
TARA	ERR598958	250	34.15	-56.97	3.40E+08	0.008	0.016	0.002	0.012	0.000	0.000
TARA	ERR598960	850	-35.19	26.29	3.88E+08	0.008	0.137	0.002	0.071	0.000	0.000
TARA	ERR598964	740	34.08	-49.82	3.88E+08	0.008	0.143	0.003	0.078	0.000	0.000
TARA	ERR598971	380	2.06	-84.55	3.24E+08	0.011	0.076	0.009	0.042	0.000	0.000
TARA	ERR598980	550	31.53	-159.02	4.08E+08	0.007	0.161	0.004	0.070	0.000	0.000
TARA	ERR598985	640	25.62	-88.45	3.28E+08	0.010	0.169	0.005	0.088	0.000	0.000
TARA	ERR598999	600	-8.97	-139.24	9.91E+07	0.006	0.084	0.004	0.041	0.000	0.000
TARA	ERR599000	800	-20.93	-35.18	3.89E+08	0.004	0.044	0.003	0.019	0.000	0.000
TARA	ERR599004	450	6.36	-103.06	8.58E+07	0.006	0.008	0.003	0.003	0.000	0.000
TARA	ERR599005	800	-8.80	-17.90	1.98E+08	0.002	0.020	0.002	0.010	0.000	0.000
TARA	ERR599008	790	-61.97	-49.50	3.32E+08	0.005	0.036	0.004	0.019	0.000	0.000
TARA	ERR599015	375	14.20	-116.64	1.01E+08	0.006	0.010	0.003	0.005	0.000	0.000
TARA	ERR599020	380	-1.89	-84.61	8.98E+07	0.028	0.135	0.023	0.076	0.000	0.001
TARA	ERR599021	1000	-29.50	37.96	8.98E+07	0.006	0.095	0.002	0.045	0.000	0.000
TARA	ERR599033	600	-8.97	-139.24	9.93E+07	0.006	0.085	0.004	0.040	0.000	0.000
TARA	ERR599034	850	-35.19	26.29	5.86E+07	0.007	0.141	0.003	0.070	0.000	0.000
TARA	ERR599044	800	-20.41	-3.16	3.34E+08	0.008	0.040	0.007	0.018	0.000	0.000
TARA	ERR599047	640	34.67	-71.29	3.08E+08	0.008	0.130	0.003	0.073	0.000	0.000
TARA	ERR599048	800	-8.80	-17.90	8.96E+07	0.002	0.021	0.002	0.009	0.000	0.000
TARA	ERR599051	450	6.36	-103.06	8.68E+07	0.006	0.008	0.003	0.003	0.000	0.000
TARA	ERR599060	450	6.36	-103.06	8.68E+07	0.006	0.007	0.003	0.003	0.000	0.000
TARA	ERR599067	380	2.06	-84.55	6.31E+07	0.011	0.079	0.009	0.042	0.000	0.000
TARA	ERR599071	488	-25.81	-111.69	3.78E+08	0.005	0.082	0.001	0.039	0.000	0.000
TARA	ERR599072	696	-23.22	-129.60	3.03E+08	0.002	0.028	0.001	0.012	0.000	0.000
TARA	ERR599076	375	14.20	-116.64	1.01E+08	0.007	0.010	0.002	0.005	0.000	0.000
TARA	ERR599083	600	-8.97	-139.24	1.02E+08	0.006	0.085	0.004	0.041	0.000	0.000
TARA	ERR599085	488	-25.81	-111.69	7.22E+07	0.005	0.077	0.002	0.038	0.000	0.000
TARA	ERR599086	350	-16.95	-100.67	4.17E+08	0.009	0.101	0.005	0.055	0.000	0.000
TARA	ERR599096	600	-8.97	-139.24	9.86E+07	0.005	0.084	0.004	0.040	0.000	0.000
TARA	ERR599112	1000	-15.34	43.29	4.35E+08	0.002	0.033	0.001	0.017	0.000	0.000
TARA	ERR599115	650	35.27	-127.73	4.38E+08	0.007	0.068	0.005	0.032	0.000	0.000
TARA	ERR599124	800	-30.15	-43.29	6.06E+07	0.004	0.086	0.002	0.036	0.000	0.000
TARA	ERR599125	790	-61.97	-49.50	7.61E+07	0.005	0.037	0.004	0.019	0.000	0.000
TARA	ERR599127	375	14.20	-116.64	1.01E+08	0.007	0.010	0.002	0.005	0.000	0.000
TARA	ERR599131	700	-31.02	4.67	5.82E+07	0.010	0.176	0.006	0.074	0.000	0.000

TARA	ERR599149	800	-20.41	-3.16	5.61E+07	0.008	0.040	0.008	0.018	0.000	0.000
TARA	ERR599152	375	14.20	-116.64	1.01E+08	0.006	0.009	0.002	0.005	0.000	0.000
TARA	ERR599154	800	-20.93	-35.18	5.92E+07	0.005	0.043	0.004	0.019	0.000	0.000
TARA	ERR599159	800	-30.15	-43.29	4.24E+08	0.004	0.080	0.002	0.035	0.000	0.000
TARA	ERR599164	1000	-29.50	37.96	1.55E+08	0.006	0.093	0.002	0.044	0.000	0.000
TARA	ERR599175	450	6.36	-103.06	8.68E+07	0.006	0.007	0.003	0.003	0.000	0.000
TARA_OMZ	ERR598946	177	-12.98	-96.02	2.29E+08	0.010	0.021	0.015	0.007	0.000	0.000
TARA_OMZ	ERR598953	177	-12.98	-96.02	9.11E+07	0.010	0.022	0.016	0.008	0.000	0.000
TARA_OMZ	ERR598981	177	-12.98	-96.02	4.47E+07	0.010	0.021	0.014	0.008	0.000	0.000
TARA_OMZ	ERR599031	600	20.85	63.59	5.86E+07	0.002	0.004	0.002	0.001	0.000	0.000
TARA_OMZ	ERR599037	270	18.73	66.39	1.95E+08	0.007	0.002	0.002	0.003	0.000	0.000
TARA_OMZ	ERR599055	480	-5.26	-85.17	4.69E+07	0.010	0.096	0.048	0.007	0.000	0.000
TARA_OMZ	ERR599062	600	20.85	63.59	3.40E+08	0.002	0.004	0.002	0.001	0.000	0.000
TARA_OMZ	ERR599109	340	19.04	64.56	7.76E+07	0.008	0.003	0.004	0.005	0.000	0.000
TARA_OMZ	ERR599128	480	-5.26	-85.17	9.47E+07	0.010	0.105	0.052	0.008	0.000	0.000
TARA_OMZ	ERR599132	480	-5.26	-85.17	2.57E+08	0.009	0.095	0.048	0.007	0.000	0.000
TARA_OMZ	ERR599166	590	39.24	-70.03	3.54E+08	0.005	0.098	0.044	0.004	0.000	0.000
TARA_OMZ	ERR599167	340	19.04	64.56	1.82E+08	0.008	0.003	0.004	0.004	0.000	0.000
MALASPINA	MP0145	4005	14.52	-26.00	1.01E+07	0.001	0.001	0.000	0.001	0.000	0.000
MALASPINA	MP0371	4003	-15.83	-33.41	2.82E+07	0.000	0.000	0.000	0.000	0.000	0.000
MALASPINA	MP0372	4003	-15.83	-33.41	3.39E+07	0.000	0.000	0.000	0.000	0.000	0.000
MALASPINA	MP0103	4005	21.51	-23.45	2.36E+07	0.000	0.000	0.000	0.000	0.000	0.000
MALASPINA	MP0203	4003	7.33	-26.00	2.27E+07	0.000	0.000	0.000	0.000	0.000	0.000
MALASPINA	MP0326	4001	-9.12	-30.19	1.72E+07	0.000	0.000	0.000	0.000	0.000	0.000
MALASPINA	MP0262	4002	-3.03	-27.33	2.61E+07	0.000	0.000	0.000	0.000	0.000	0.000
MALASPINA	MPI1371	-	-39.24	135.14	2.13E+07	0.000	0.000	0.000	0.000	0.000	0.000
MALASPINA	MP0900	4002	-33.55	39.89	1.97E+07	0.000	0.000	0.000	0.000	0.000	0.000
MALASPINA	MP0901	4002	-33.55	39.89	2.07E+07	0.000	0.000	0.000	0.000	0.000	0.000
MALASPINA	MP0740	4001	-31.81	6.84	2.15E+07	0.000	0.001	0.001	0.000	0.000	0.000
MALASPINA	MP0758	3902	-32.81	12.77	1.64E+07	0.000	0.000	0.000	0.000	0.000	0.000
MALASPINA	MP0759	3902	-32.81	12.77	2.26E+07	0.001	0.000	0.001	0.001	0.000	0.000
MALASPINA	MP0555	3199	-26.91	-21.43	2.10E+07	0.000	0.000	0.000	0.000	0.000	0.000
MALASPINA	MP0556	3199	-26.91	-21.43	2.06E+07	0.000	0.000	0.000	0.000	0.000	0.000
MALASPINA	MP0626	3850	-28.62	-11.80	2.09E+07	0.000	0.000	0.000	0.000	0.000	0.005
MALASPINA	MP0739	4001	-31.81	6.84	8.94E+06	0.000	0.000	0.000	0.000	0.000	0.000
MALASPINA	MP0959	3505	-27.98	63.25	2.40E+07	0.000	0.000	0.000	0.000	0.000	0.006
MALASPINA	MP1092	4000	-29.81	82.62	1.84E+07	0.000	0.000	0.000	0.000	0.000	0.000
MALASPINA	MP1140	2402	-29.65	92.99	2.07E+07	0.000	0.000	0.000	0.000	0.000	0.000
MALASPINA	MP1141	2402	-29.65	92.99	2.67E+07	0.000	0.000	0.000	0.000	0.000	0.000
MALASPINA	MP1202	4001	-30.33	103.31	2.45E+07	0.000	0.000	0.000	0.000	0.000	0.000
MALASPINA	MP1374	3996	-39.23	135.19	1.89E+07	0.000	0.000	0.001	0.000	0.000	0.000
MALASPINA	MP1896	4013	21.06	-150.35	2.67E+07	0.000	0.000	0.000	0.000	0.000	0.000
MALASPINA	MP1434	4001	-38.64	150.41	2.09E+07	0.000	0.000	0.000	0.000	0.000	0.000
MALASPINA	MP1482	3501	-28.41	179.14	2.13E+07	0.000	0.000	0.000	0.000	0.000	0.000

MALASPINA	MP1483	3501	-28.41	179.14	2.24E+07	0.000	0.001	0.000	0.000	0.000	0.000
MALASPINA	MP1493	2148	-25.49	-179.52	2.24E+07	0.000	0.000	0.000	0.000	0.000	0.000
MALASPINA	MP1648	4018	-5.74	-170.77	1.71E+07	0.000	0.000	0.000	0.000	0.000	0.000
MALASPINA	MP1649	4018	-5.74	-170.77	2.61E+07	0.000	0.000	0.000	0.000	0.000	0.000
MALASPINA	MP2016	4004	18.04	-133.26	2.25E+07	0.000	0.000	0.000	0.000	0.000	0.000
MALASPINA	MP2052	4002	15.91	-124.49	1.79E+07	0.000	0.000	0.000	0.000	0.000	0.000
MALASPINA	MP2158	3103	12.00	-108.06	2.10E+07	0.000	0.000	0.000	0.000	0.000	0.000
MALASPINA	MP2159	3103	12.00	-108.06	2.19E+07	0.000	0.000	0.001	0.000	0.003	0.002
MALASPINA	MP2252	3008	10.09	-99.25	2.53E+07	0.000	0.000	0.000	0.000	0.000	0.000
MALASPINA	MP2253	3008	10.09	-99.25	2.39E+07	0.000	0.001	0.001	0.000	0.001	0.001
MALASPINA	MP2914	4003	29.97	-23.69	3.07E+07	0.000	0.000	0.000	0.000	0.000	0.000
MALASPINA	MP2633	4003	20.00	-52.63	3.78E+07	0.000	0.000	0.000	0.000	0.000	0.000
MALASPINA	MP2634	4003	20.00	-52.63	3.93E+07	0.000	0.000	0.000	0.000	0.000	0.000
MALASPINA	MP2968	4003	32.08	-17.26	2.74E+07	0.000	0.000	0.000	0.000	0.000	0.000

Table S7. Digital Protologue for *Candidatus* Thioglobus vadi, *Candidatus* Thioglobus vulcanius and *Candidatus* Thioglobus plumae.

**Genus name**	*Candidatus* Thioglobus	*Candidatus* Thioglobus	*Candidatus* Thioglobus
**Species name**	*Candidatus* Thioglobus vadi	*Candidatus* Thioglobus vulcanius	*Candidatus* Thioglobus plumae
**Specific epithet**	vadi	vulcanius	plumae
**Species status**	sp. nov.	sp. nov.	sp. nov.
**Status**	uncultivated	uncultivated	uncultivated
**Species etymology**	Thioglobus vadi (va'di. L. gen. n. *vadi* of a shallow place).	Thioglobus volcanius (vol.ca'ni.us. N.L. masc. adj. volcanius belonging to Volcanus, belonging to a volcanic area). See as precedents Bremerella volcania, Thermoplasma volcanium.	Thioglobus plumae (plu'mae. L. gen. n. plumae of a 'feather' of a plume).
**Description of the new taxon and diagnostic traits**	*Candidatus* Thioglobus vadi is highly abundant in mesopelagic plume of Macauley volcano, Kermadec Arc (South Pacific Ocean). Additionally, it persists in the surface and deep chlorophyll maximum layer of the open-ocean. Genome annotation predicts chemolithoautotrophy fueled by reduced sulfur species with oxygen as electron acceptor. Dark carbon fixation is conducted via Calvin Benson Bassham cycle. The high expression of cytochromes could be potentially linked to iron oxidation.	*Candidatus* Thioglobus vulcanius is abundant in the plume of the cone of Brothers bathypelagic volcano, Kermadec Arc (South Pacific Ocean). Genome annotation indicates chemolithoautotrophy and supports the oxidation of reduced sulfur compounds with oxygen as electron acceptor. Dark carbon fixation is conducted via Calvin Benson Bassham cycle. The genomes harbor a high number of iron acquisition genes. It could potentially be specialized to highly variable iron and sulfide concentrations. The expression profile exhibits a high expression of resistance genes.	*Candidatus* Thioglobus plumae is abundant in the plume of the NWC of Brothers bathypelagic volcano, Kermadec Arc (South Pacific Ocean). Genome annotation indicates chemolithoautotrophy and supports the oxidation of reduced sulfur compounds with oxygen as electron acceptor. Dark carbon fixation is conducted via Calvin Benson Bassham cycle. It is shown to be present and abundant in other sulfur-rich studied plumes.
**Country of origin**	New Zealand	New Zealand	New Zealand
**Region of origin**	Kermadec arc, Macauley volcano	Kermadec arc, Brothers volcano, cone	Kermadec arc, Brothers volcano, NWC

**Source of isolation**	Hydrothermal plume	Hydrothermal plume	Hydrothermal plume
**Sampling date (dd/mm/yyyy)**	27/12/2016	08/01/2017	09/01/2017
**Latitude (xx°xx′xx″N/S)**	-30.212934	-34.8762350	-34.8694167
**Longitude (xx°xx′xx″E/W)**	-178.449336	179.0753393	179.0626500
**Depth (m)**	275	1307.8	1560.3
**Genome accession number**	ERS6225882	ERS6225884	ERS6225886
**Genome status**	incomplete	incomplete	incomplete
**Genome size**	985958	1131004	1013498
**GC mol%**	38.37	38.16	39.62

Table S8. Plume intensity based on ΔNTU and SUP05 relative abundance in 054CTD 16S rRNA amplicon data.

	ΔNTU	CTT
**54CTD_b4**	0.0466	28%
**54CTD_b8**	0.1222	40%
**54CTD_b12**	0.0475	18%
**54CTD_b15**	0.0025	4%
**54CTD_b18**	0.0124	5%

Supplementary figures

Figure S1. Phylogenetic tree of the SUP05 clade based on 16S rRNA gene sequences. This tree was calculated based on 60 long sequences using PhyML, a 30% sequence conservation filter, and 100 bootstraps. Phyloflash [13] reconstructed 16S rRNA gene sequences are depicted in orange (>900 bp), 16S rRNA genes retrieved from MAGs are given in green and 16S rRNA amplicon sequences in blue. The bar indicates 10% estimated sequence changes.

Figure S2. Distance-based redundancy analysis (dbRDA) of microbial communities in McV (A) and BrV (B). This analysis was calculated based on a Bray-Curtis dissimilarity matrix of standardized log environmental parameters. The Bray-Curtis matrix was calculated based on the relative abundance of 16S rRNA gene sequences with a 1% abundance threshold. Non-parametric permutational multivariate analysis of variance (PerMANOVA; ref. 42) results were calculated using the "Adonis" function of the vegan package in R [52]. CTA – *Candidatus* Thioglobus autotrophicus-related sequences; CTT – *Candidatus* Thioglobus thermophilus-related sequences and CPS – *Candidatus* Pseudothioglobus singularis-related sequences.

Figure S3. Gibb's free energy available from the oxidation of one electron of H_2S and FeOx. H_2S concentrationis modelled using the highest H_2S and FeOx concentration in the fluid and DFe plume concentrations. All processes are considered to be aerobic as oxygen is present across all samples. Samples are ranked based on the measured ΔNTU from highest to lowest.

Figure S4. Total cell counts and relative abundance of selected microbial taxa. A) Total cell counts determinedby counting DAPI stained cells, whereas SUP05 cells were targeted with a SUP05 specific probe (SUP05_1241) and counted. B) Abundance of the microbial groups counted relative to DAPI stained cells. Samples are ranked as in figure 2.

Figure S5. Relative abundance of sorted 16S rRNA genes from metagenomes. 16S rRNA genes were

sorted using SortMeRNA [11] and taxonomically assigned based on SILVA SSU132 taxonomy. The relative abundance shown in this graph has a 1% cut-off. The dendrogram on the upper part of the graph depicts the result of a complete linkage hierarchical clustering based on a Bray Curtis dissimilarity matrix of the community composition.

Figure S6. Functional comparison between plume and background metagenomes. A) Neighbor-Joining (NJ) tree based on functional genes of 13 metagenomes: McV, BrV, Woody Crack (MAR - Mid-Atlantic Ridge) and Mariner, Kilo Moana (Eastern Lau Spreading Centre - ESCL) and South Pacific Background samples (SAMN07136823 - 201 m depth and SAMN07136798 - 1023 m depth). Genes were analyzed by DIAMOND blast [9] against the UniRef100 database and meganized using MEGAN (default settings) [10]. B) Principal Coordinates Analysis (PCoA) using Bray-Curtis dissimilatory matrix in three dimensions based on functional profiles of 13 metagenomes. Bi-plot implementation depicts the functional groups that contribute the most to the variation, represented by vectors that reveal the direction of the increase.

Figure S7. Comparison of normalized numbers of functional genes in 13 metagenomes. The metagenomes originate from McV, BrV, Woody Crack (MAR - Mid-Atlantic Ridge) and Mariner, Kilo Moana (Eastern Lau Spreading Centre - ESCL) and South Pacific Ocean Background (SAMN07136823 - 201 m depth and SAMN07136798 - 1023 m depth). The number of genes for cytochrome b is plotted on the secondary axis. Genes were compared against the UniRef100 database using DIAMOND v 0.8.24.86 [9] and meganized with MEGAN v6.6.0 [10]. Genes compared are: SOX - Sulfur-oxidizing genes, *dsr*A – dissimilatory sulfite reductaseA, cytochrome genes and DNA polymerase III.

Figure S8. Taxonomic affiliation of bacterial cytochrome genes across McV, BrV and Mariner and Kilo Moana (Eastern Lau Spreading Centre - ESLC) metagenomes. The barchart depicts the relative abundance of the cytochrome genes affiliated to SUP05, SAR11, and other Bacteria. Cytochrome genes were analysed with MEGAN 6.6.0 [10] using Lowest Common Ancestor (LCA) assignment algorithm.

Figure S9. Metabolic potential of non-SUP05 MAGs with a completeness of >50%. Bacterial genomes were manually screened for genes for: - sulfur metabolism (sox – sulfur oxidizing enzyme; Sqr – sulfide:quinone oxidoreductase; Fcc – flavocytochrome c; Dsr – dissimilatory sulfite reductase; Apr – dissimilatory adenylylsulfate reductase), hydrogen and carbon monoxide oxidation, carbon fixation pathways (rTCA-reverse tricarboxylic acid cycle, reductive acetyl-CoA and CBB – Calvin Benson Bassham cycle), nitrogen fixation and nitrogen reduction (NAP – nitrate reductase; NIR – nitrite reductase; NOR – nitric oxide reductase, NOS – nitrous oxide reductase), transporters and lastly motility genes.

Figure S10. Distance-based redundancy analysis (dbRDA) of SUP05 MAGs. This analysis was calculated based on a Bray-Curtis dissimilarity matrix of MAGs' ANI values and standardized log environmental parameters.

Figure S11. **Relative abundance of the 30 most expressed genes of the most complete SUP05 MAGs (A and B) and putatively heterotrophic MAGs (C).** The relative abundance is given as log normalized transcript per million (TPM). A) Metatranscriptomes of McV were mapped with 97% identity to MAG-1 and MAG-2. B) Metatranscriptomes of BrV were mapped (97% identity) to MAG-5 and MAG-6. The patterns of the bars link the expression of a specific gene to the metatranscriptome and the coupled-bar represents the same gene in two metatranscriptomes. C) MAG-7_1 to 3, were mapped (97% identity) to metatranscriptomes of BrV and are given as a sunburst plot. Genes in the SUP05 MAGs, such as house-keeping genes, carbon metabolism, sulfur-oxidation pathway (SOX), cytochromes, nitrogen metabolism and transporters, are represented by different colors.

Figure S12. **Expression ratio between cytochrome, sox and house-keeping genes in six SUP05 MAGs across four metatranscriptomes from McV and BrV.** SUP05-1-4 is the average of expression rate in MAG-1 to MAG-4. House-keeping genes analysed are *pro*C, *rec*A and *rpo*D. House-keeping gene expression is given on the secondary axis for activity comparison between MAGs and metatranscriptomes.

Figure S13. Abundance of SUP05-related MAGs in the surface metagenomes of TARA OCEAN (PRJEB1787), seven plume metagenomes of Lau Basin (>1900 m depth; Tai Malila, Tahi Moana, Mariner, Kilo Moana, Abe) and Mid-Atlantic Ridge (Woody Crack - 828 m depth). Reads were mapped to the SUP05 clusters using BBMap [27] with 99% minimum identity. Abundance was calculated as RPKM.

Figure S14. Abundance of SUP05-related MAGs in the deep chlorophyll maximum (DCM) metagenomes of TARA Ocean (PRJEB1787). SUP05 MAG-1 to 4 are *Candidatus* Thioglobus vadi strains. Reads were mapped unambiguously to the SUP05 clusters using BBmap [27] with 99% minimum identity. Abundance was calculated as RPKM.

Figure S15. Expression of genes for heavy-metal transporter/detoxification proteins in SUP05 MAGs.

References

1. Standard methods for the examination of water and wastewater, 16th ed. 1985. American Public Health Association, Washington, D.C.

2. Walker SL, Baker ET, Resing JA, Nakamura K, McLain PD. A new tool for detecting hydrothermal plumes: an ORP sensor for the PMEL MAPR. AGU Fall Meeting Abstracts. 2007;V21D-0753 (abstract).

3. Baker ET, Resing JA, Haymon RM, Tunnicliffe V, Martinez F, Ferrini V, et al. How many vent fields? New estimates of vent field populations on ocean ridges from precise mapping of hydrothermal discharge locations. Prog Earth Planet Sci. 2016;449:186-96.

4. Herlemann DPR, Labrenz M, Jürgens K, Bertilsson S, Waniek JJ, Andersson AF. Transitions in bacterial communities along the 2000 km salinity gradient of the Baltic Sea. ISME J. 2011;5(10):1571–9.

5. Reintjes G, Tegetmeyer HE, Bürgisser M, Orlić S, Tews I, Zubkov M, et al. On-site analysis of bacterial communities of the ultraoligotrophic South Pacific Gyre. Appl Environ Microbiol. 2019;85(14):e00184-19.

6. Anantharaman K, Breier JA, Dick GJ. Metagenomic resolution of microbial functions in deep-sea hydrothermal plumes across the Eastern Lau Spreading Center. ISME J. 2016;10(1):225–39.

7. Meier D V., Bach W, Girguis PR, Gruber-Vodicka HR, Reeves EP, Richter M, et al. Heterotrophic Proteobacteria in the vicinity of diffuse hydrothermal venting. Environ Microbiol. 2016;18(12):4348–68.

8. Biller SJ, Berube PM, Dooley K, Williams M, Satinsky BM, Hackl T, et al. Data descriptor: Marine microbial metagenomes sampled across space and time. Sci Data. 2018;5(1):1–7.

9. Buchfink B, Xie C, Huson DH. Fast and sensitive protein alignment using DIAMOND. Nat methods.

2015;12(1):59-60.

10. Huson DH, Albrecht B, Bağci C, Bessarab I, Górska A, Jolic D, et al. MEGAN-LR: New algorithms allow accurate binning and easy interactive exploration of metagenomic long reads and contigs.Biol Direct. 2018;13(1):1-7.

11. Kopylova E, Noé L, Touzet H. SortMeRNA: Fast and accurate filtering of ribosomal RNAs in metatranscriptomic data. Bioinformatics. 2012;28(24):3211-7.

12. Quast C, Pruesse E, Yilmaz P, Gerken J, Schweer T, Yarza P, et al. The SILVA ribosomal RNA gene database project: Improved data processing and web-based tools. Nucleic Acids Res. 2013;41(D1).

13. Gruber-Vodicka HR, Seah BKB, Pruesse E. phyloFlash: Rapid small-subunit rRNA profiling and targeted assembly from metagenomes. mSystems. 2020;5(5):e00920-20.

14. Eren AM, Kiefl E, Shaiber A, Veseli I, Miller SE, Schechter MS, et al. Community-led, integrated, reproducible multi-omics with anvi'o. Nat Microbiol. 2021;6(1):3-6.

15. Parks DH, Imelfort M, Skennerton CT, Hugenholtz P, Tyson GW. CheckM: Assessing the quality of microbial genomes recovered from isolates, single cells, and metagenomes. Genome Res. 2015;25(7):1043–55.

16. Meier D V., Pjevac P, Bach W, Hourdez S, Girguis PR, Vidoudez C, et al. Niche partitioning of diverse sulfur-oxidizing bacteria at hydrothermal vents. ISME J. 2017;11(7):1545–58.

17. Eddy SR. Accelerated profile HMM searches. PLoS Comput Biol. 2011;7(10).

18. Chaumeil P-A, Mussig AJ, Hugenholtz P, Parks DH. GTDB-Tk: a toolkit to classify genomes with the Genome Taxonomy Database. Bioinformatics. 2019;36(6):1925–7.

19. Letunic I, Bork P. Interactive Tree Of Life (iTOL) v5: an online tool for phylogenetic tree display and annotation. Nucleic Acids Res. 2021;49:W293-W296.

20. Richter M, Rosselló-Móra R, Oliver Glöckner F, Peplies J. JSpeciesWS: A web server for prokaryotic species circumscription based on pairwise genome comparison. Bioinformatics. 2015;32(6):929–31.

21. Aziz RK, Bartels D, Best A, DeJongh M, Disz T, Edwards RA, et al. The RAST Server: Rapid annotations using subsystems technology. BMC Genomics. 2008;9.

22. Seemann T. Prokka: Rapid prokaryotic genome annotation. Bioinformatics. 2014;30(14):2068-9.

23. Lombard V, Golaconda Ramulu H, Drula E, Coutinho PM, Henrissat B. The carbohydrate-active enzymes database (CAZy) in 2013. Nucleic Acids Res. 2014;42(D1):D490.

24. Garber AI, Nealson KH, Okamoto A, McAllister SM, Chan CS, Barco RA, et al. FeGenie: A comprehensive tool for the identification of Iron genes and iron gene neighborhoods in genomeand metagenome assemblies. Front Microbiol. 2020;11:37.

25. Guo J, Bolduc B, Zayed AA, Varsani A, Dominguez-Huerta G, Delmont TO, et al. VirSorter2: a multi-classifier, expert-guided approach to detect diverse DNA and RNA viruses. Microbiome. 20219(1):1–13.

26. Duarte CM. Seafaring in the 21st century: The Malaspina 2010 circumnavigation expedition. Limnol Oceanogr Bull. 2015;24:11-14.

27. Bushnell B. BBMap (version 35.14) [Software]. Available at https://sourceforge.net/projects/bbmap/. 2015.

28. Pruesse E, Peplies J, Glöckner FO. SINA: Accurate high-throughput multiple sequence alignmentof ribosomal RNA genes. Bioinformatics. 2012;28(14):1823–9.

29. Ludwig W, Strunk O, Westram R, Richter L, Meier H, Yadhukumar A, et al. ARB: A software environment for sequence data. Nucleic Acids Res. 2004;32(4):1363–71.

30. Guindon S, Dufayard JF, Lefort V, Anisimova M, Hordijk W, Gascuel O. New algorithms and methods to estimate maximum-likelihood phylogenies: Assessing the performance of PhyML 3.0.Syst Biol. 2010;59(3):307–21.

31. Stamatakis A. RAxML version 8: A tool for phylogenetic analysis and post-analysis of large phylogenies. Bioinformatics. 2014;30(9):1312–3.

32. Shah V, Morris RM. Genome sequence of "*Candidatus* Thioglobus autotrophica" strain EF1, a chemoautotroph from the SUP05 clade of marine Gammaproteobacteria. Genome Announc. 2015;3(5):e01156-15.

33. Ponnudurai R, Sayavedra L, Kleiner M, Heiden SE, Thürmer A, Felbeck H, et al. Genome sequence of the sulfur-oxidizing *Bathymodiolus thermophilus* gill endosymbiont. Stand Genomic Sci. 2017;12(1):1-9.

34. Marshall KT, Morris RM. Isolation of an aerobic sulfur oxidizer from the SUP05/Arctic96BD-19 clade. ISME J. 2013;7(2):452–5.

35. Pernthaler A, Pernthaler J, Amann R. Fluorescence in situ hybridization and catalyzed reporter deposition for the identification of marine bacteria. Appl Environ Microbiol. 2002;68(6):3094–101.

36. Amann RI, Binder BJ, Olson RJ, Chisholm SW, Devereux R, Stahl DA. Combination of 16S rRNA-targeted oligonucleotide probes with flow cytometry for analyzing mixed microbial

populations.Appl Environ Microbiol. 1990;56(6):1919–25.

37. Daims H, Brühl A, Amann R, Schleifer KH, Wagner M. The domain-specific probe EUB338 is insufficient for the detection of all bacteria: Development and evaluation of a more comprehensive probe set. Syst Appl Microbiol. 1999;22(3):434–44.

38. Wallner G, Amann R, Beisker W. Optimizing fluorescent *in situ* hybridization with rRNA-targeted oligonucleotide probes for flow cytometric identification of microorganisms. Cytometry. 1993;14(2):136–43.

39. Stahl D. A., Amann R. Development and application of nucleic acid probes in bacterial systematics. In: Stackebrandt E, Goodfellow M (eds). Nucleic Acid Techniques in Bacterial Systematics. 1991. John Wiley & Sons Ltd., Chichester, pp 205–248.

40. Manz W, Amann R, Ludwig W, Wagner M, Schleifer KH. Phylogenetic oligodeoxynucleotide probes for the major subclasses of Proteobacteria: Problems and Solutions. Syst Appl Microbiol. 1992;15(4):593–600.

41. Kleint C, Bach W, Diehl A, Fröhberg N, Garbe-Schönberg D, Hartmann JF, et al. Geochemical characterization of highly diverse hydrothermal fluids from volcanic vent systems of the Kermadec intraoceanic arc. Chem Geol. 2019;528:119289.

42. Anderson MJ. A new method for non parametric multivariate analysis of variance. Austral Ecol. 2001;26:32–46.

43. R Core Team. R: A language and environment for statistical computing. 2013. R Foundation for Statistical Computing, Vienna, Austria.

44. Pena EA, Slate EH. gvlma: Global validation of linear models assumptions. R package version 1.0.0.3. https://CRAN.R-project.org/package=gvlma. 2019.

45. Clarke KR. Non-parametric multivariate analysis of changes in community structure. Austral Ecol. 1993;18:117–43.

46. Dittmar T, Koch B, Hertkorn N, Kattner G. A simple and efficient method for the solid-phase extraction of dissolved organic matter (SPE-DOM) from seawater. Limnol Oceanogr Methods. 2008;6(6):230-5.

47. Hansen CT, Niggemann J, Giebel HA, Simon M, Bach W, Dittmar T. Biodegradability of hydrothermally altered deep-sea dissolved organic matter. Mar Chem. 2019;217:103706.

48. Neuholz R, Kleint C, Schnetger B, Koschinsky A, Laan P, Middag R, et al. Submarine hydrothermal discharge and fluxes of dissolved Fe and Mn, and He isotopes at Brothers Volcano based on radium isotopes. Minerals. 2020;10(11):969.

49. Schnetger B, Lehners C. Determination of nitrate plus nitrite in small volume marine water samples using vanadium(III)chloride as a reduction agent. Mar Chem. 2014;160:91–8.

50. O`Dell JW. Method 365.1, Determination of phosphorus by semi-automated colorimetry. EPA—USA Prot Agency. 1993:1-5.

51. Goris J, Konstantinidis KT, Klappenbach JA, Coenye T, Vandamme P, Tiedje JM. DNA-DNA hybridization values and their relationship to whole-genome sequence similarities. Int J Syst EvolMicrobiol. 2007;57(1):81–91.

52. Oksanen J, Blanchet FG, Friendly M, Kindt R, Legendre P, McGlinn D, et al. Vegan: Community ecology package. 2020.

53. Zhou Z, Tran PQ, Kieft K, Anantharaman K. Genome diversification in globally distributed novel marine Proteobacteria is linked to environmental adaptation. ISME J. 2020;14(8):2060–77.
