# Supplementary material for: Niche differentiation of sulfur-oxidizing bacteria (SUP05) in submarine hydrothermal plumes
Source: ISME J. 2022 Jan 26;16(6):1479–90. doi: 10.1038/s41396-022-01195-x (PMC9123188; doi:10.1038/s41396-022-01195-x)
Supplement: Supplementary file 2 — Supplementary Figures [file 41396_2022_1195_MOESM2_ESM.pdf]

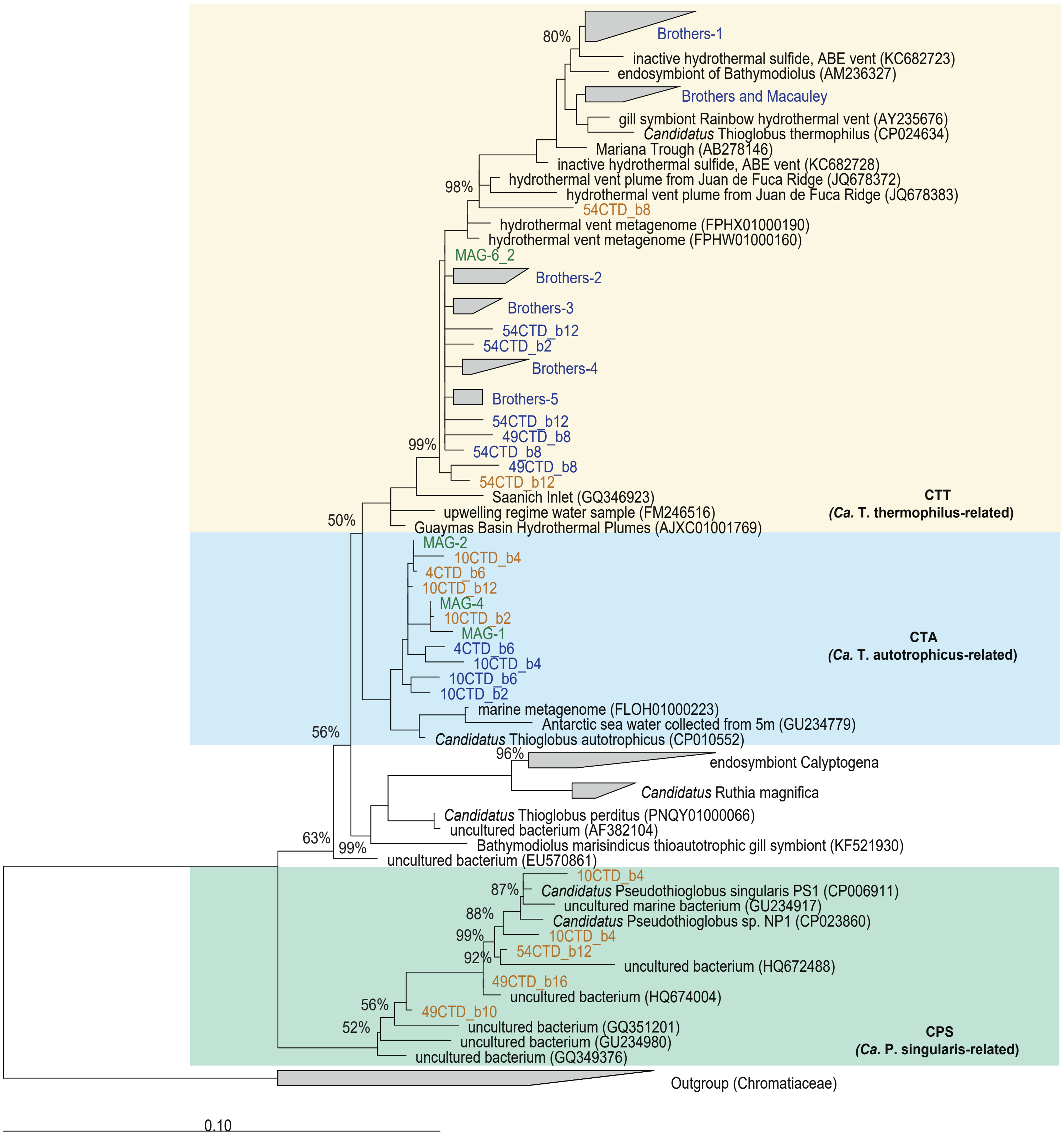


**Figure S1. Phylogenetic tree of the SUP05 clade based on 16S rRNA gene sequences. T**his tree was calculated based on 60 long sequences using PhyML, a 30% sequence conservation filter, and 100 bootstraps. Phyloflash [13] reconstructed 16S rRNA gene sequences are depicted in orange (>900 bp), 16S rRNA genes retrieved from MAGs are given in green and 16S rRNA amplicon sequences in blue. The bar indicates 10% estimated sequence changes.

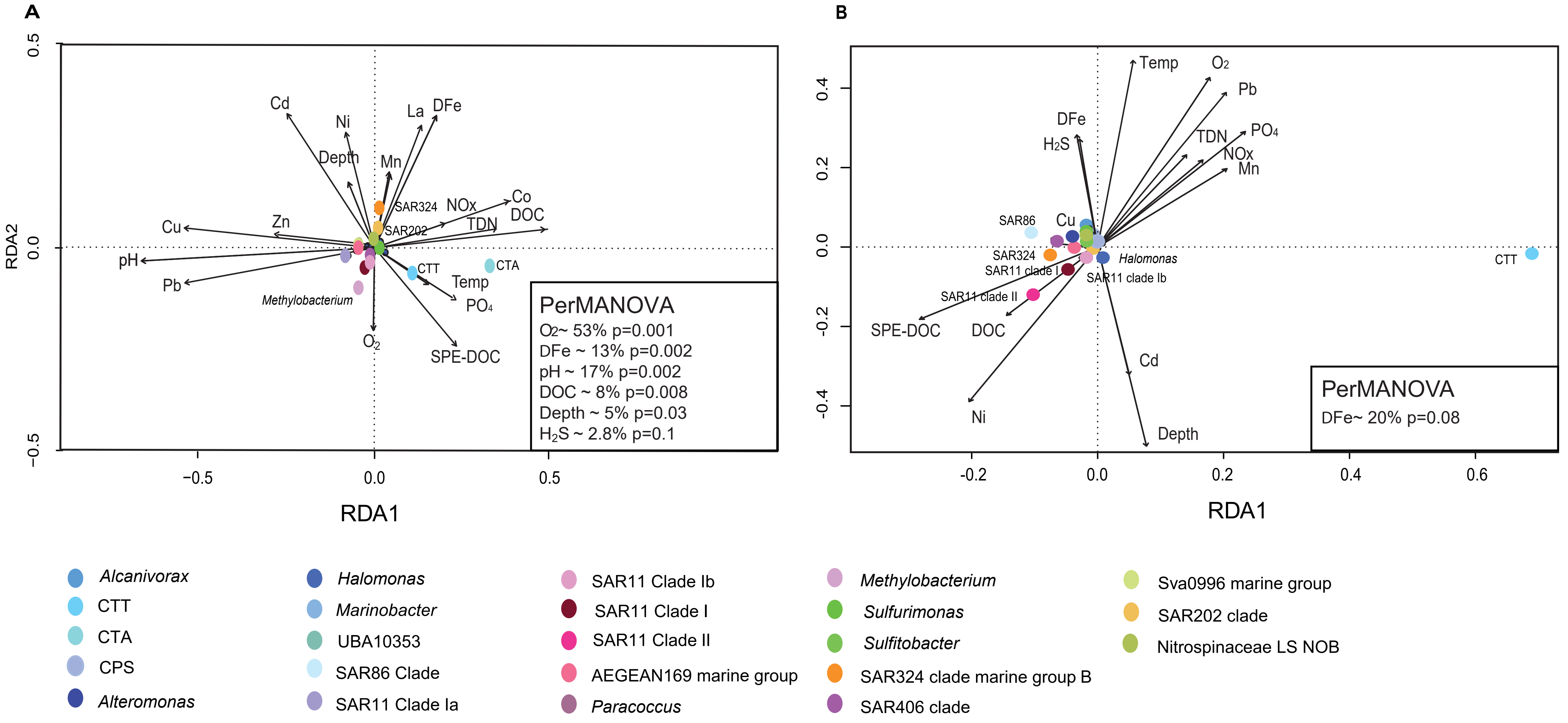


**Figure S2. Distance‐based redundancy analysis (dbRDA) of microbial communities in McV (A) and BrV (B).** This analysis was calculated based on a Bray‐Curtis dissimilarity matrix of standardized log environmental parameters. The Bray‐Curtis matrix was calculated based on the relative abundance of 16S rRNA gene sequences with a 1% abundance threshold. Non‐parametric permutational multivariate analysis of variance (PerMANOVA; ref. 42) results were calculated using the “Adonis” function of the vegan package in R [52]. CTA – *Candidatus* Thioglobus autotrophicus‐related sequences; CTT – *Candidatus* Thioglobus thermophilus‐related sequences and CPS – *Candidatus* Pseudothioglobus singularis‐related sequences.

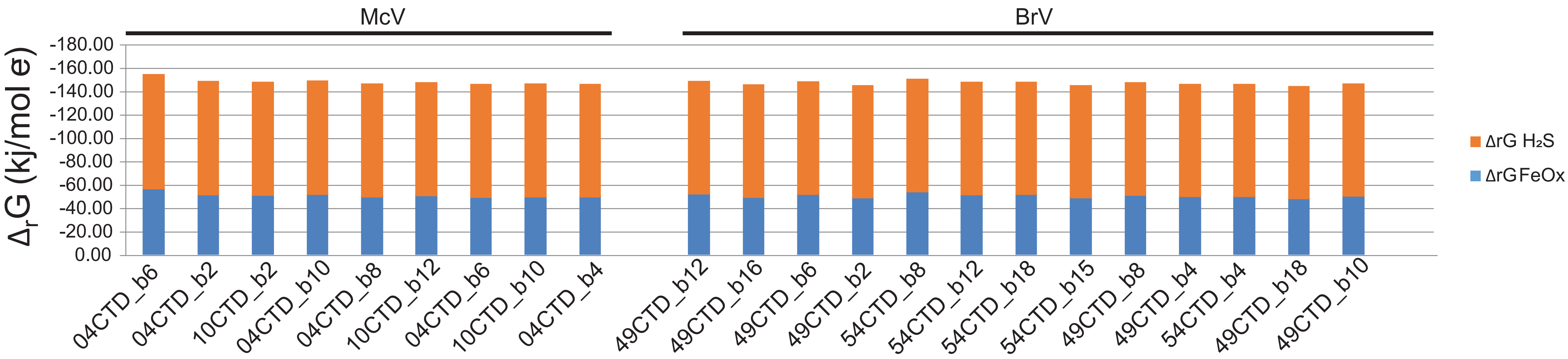


**Figure S3. Gibb's free energy available from the oxidation of one electron of $H_2S$ and FeOx.** $H_2S$ concentration is modelled using the highest $H_2S$ and FeOx concentration in the fluid and DFe plume concentrations. All processes are considered to be aerobic as oxygen is present across all samples. Samples are ranked based on the measured ΔNTU from highest to lowest.

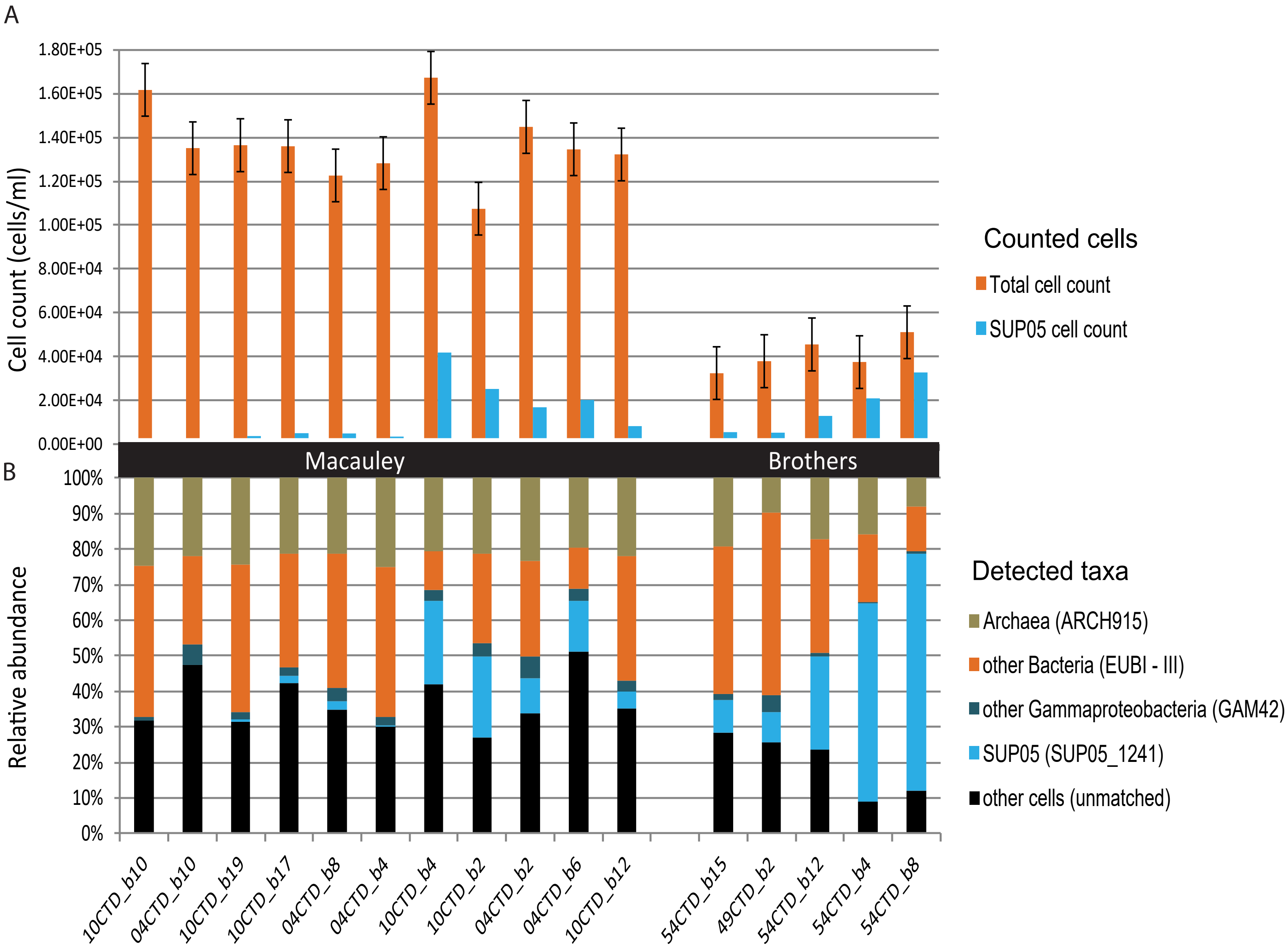


**Figure S4. Total cell counts and relative abundance of selected microbial taxa.** A) Total cell counts determined by counting DAPI stained cells, whereas SUP05 cells were targeted with a SUP05 specific probe (SUP05_1241) and counted. B) Abundance of the microbial groups counted relative to DAPI stained cells. Samples are ranked as in figure 2.

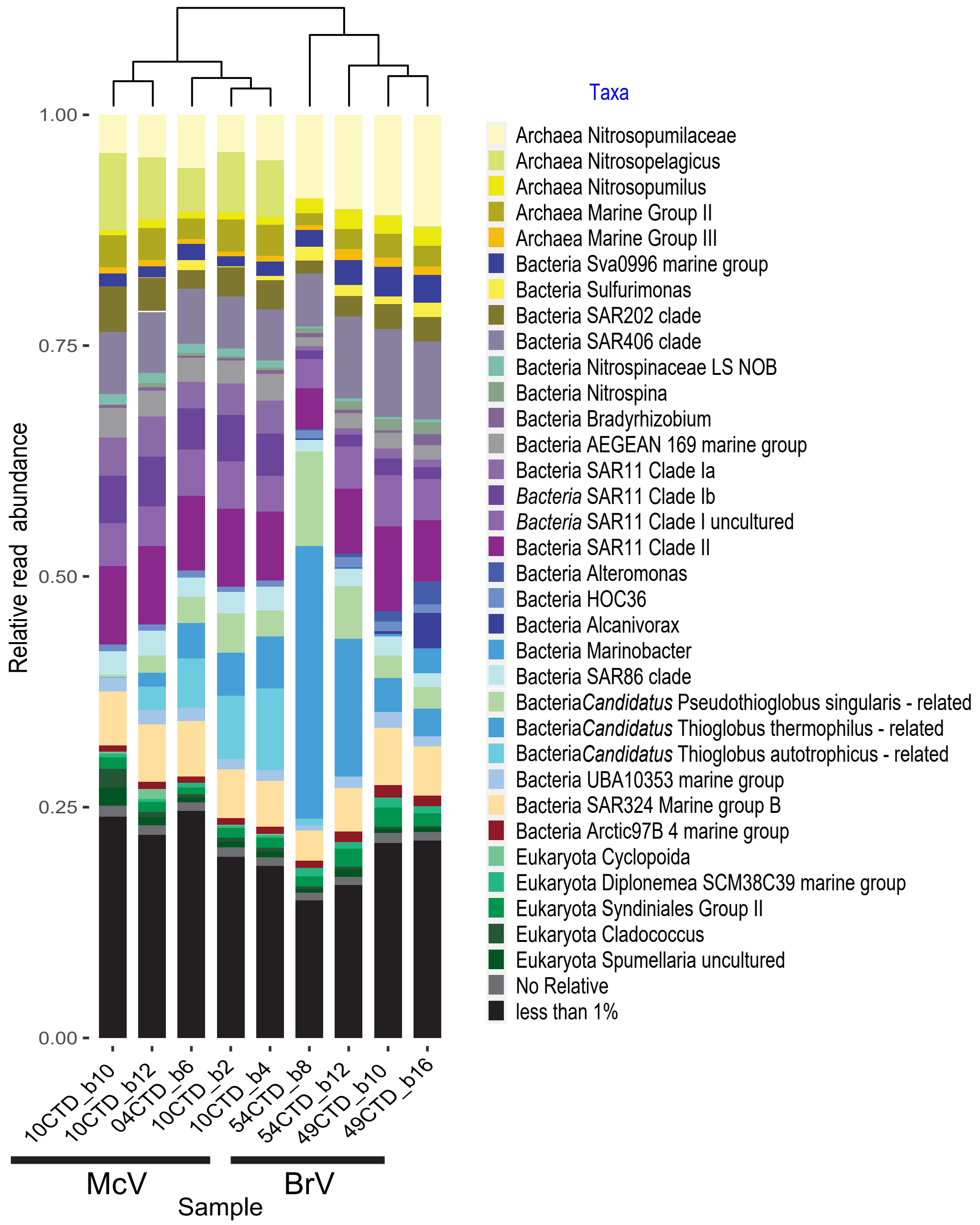


**Figure S5. Relative abundance of sorted 16S rRNA genes from metagenomes.** 16S rRNA genes were sorted using SortMeRNA [11] and taxonomically assigned based on SILVA SSU132 taxonomy. The relative abundance shown in this graph has a 1% cut-off. The dendrogram on the upper part of the graph depicts the result of a complete linkage hierarchical clustering based on a Bray Curtis dissimilarity matrix of the community composition.

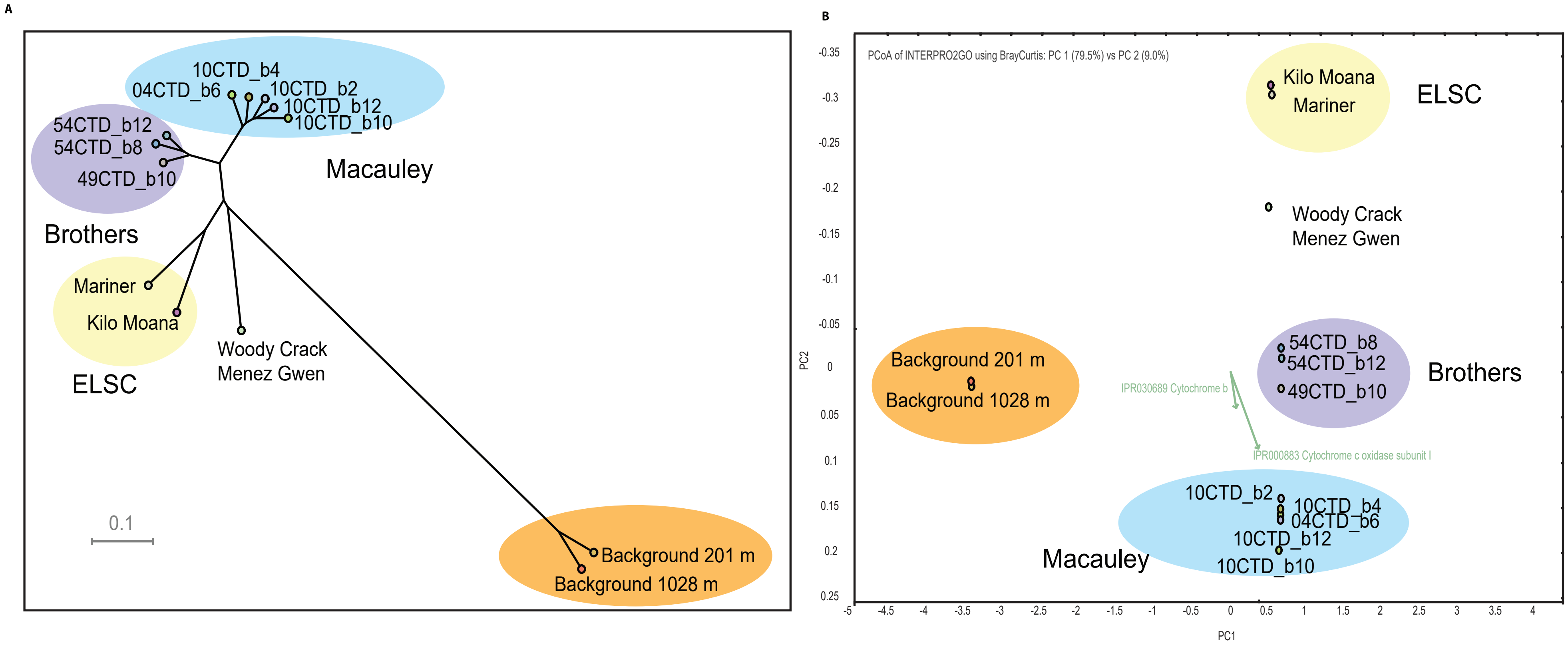


**Figure S6. Functional comparison between plume and background metagenomes.** A) Neighbor - Joining (NJ) tree based on functional genes of 13 metagenomes: McV, BrV, Woody Crack (MAR - Mid - Atlantic Ridge) and Mariner, Kilo Moana (Eastern Lau Spreading Centre - ESCL) and South Pacific Background samples (SAMN07136823 - 201 m depth and SAMN07136798 - 1023 m depth). Genes were analyzed by DIAMOND blast [9] against the UniRef100 database and meganized using MEGAN (default settings) [10]. B) Principal Coordinates Analysis (PCoA) using Bray - Curtis dissimilatory matrix in three dimensions based on functional profiles of 13 metagenomes. Bi - plot implementation depicts the functional groups that contribute the most to the variation, represented by vectors that reveal the direction of the increase.

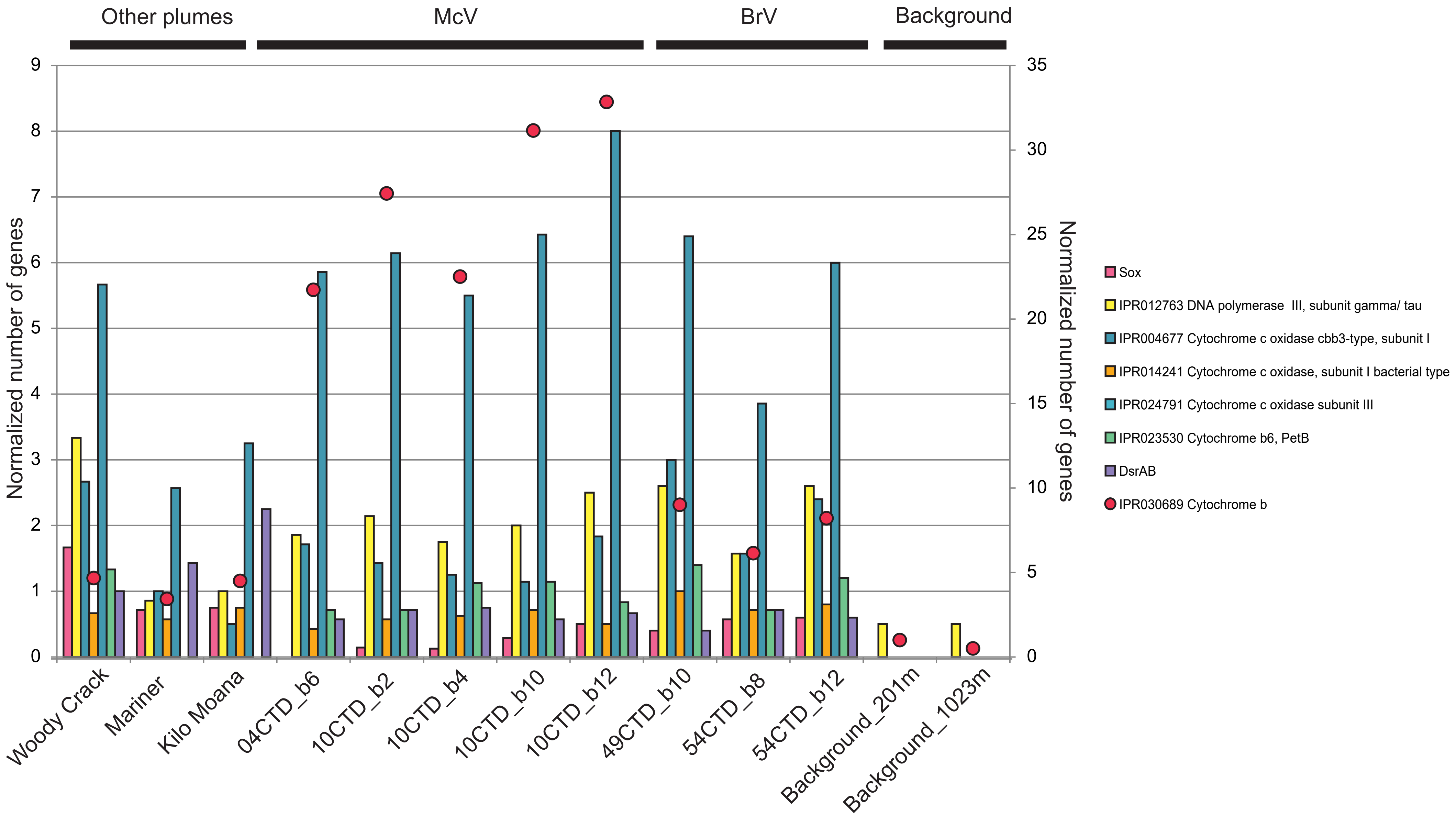


**Figure S7. Comparison of normalized numbers of functional genes in 13 metagenomes.** The metagenomes originate from McV, BrV, Woody Crack (MAR - Mid - Atlantic Ridge) and Mariner, KiloMoana (Eastern Lau Spreading Centre - ESCL) and South Pacific Ocean Background (SAMN07136823 - 201 m depth and SAMN07136798 - 1023 m depth). The number of genes for cytochrome b is plotted on the secondary axis. Genes were compared against the UniRef100 database using DIAMOND v0.8.24.86 [9] and meganized with MEGAN v6.6.0 [10]. Genes compared are: SOX - Sulfur - oxidizing genes, dsrA – dissimilatory sulfite reductase A, cytochrome genes and DNA polymerase III.

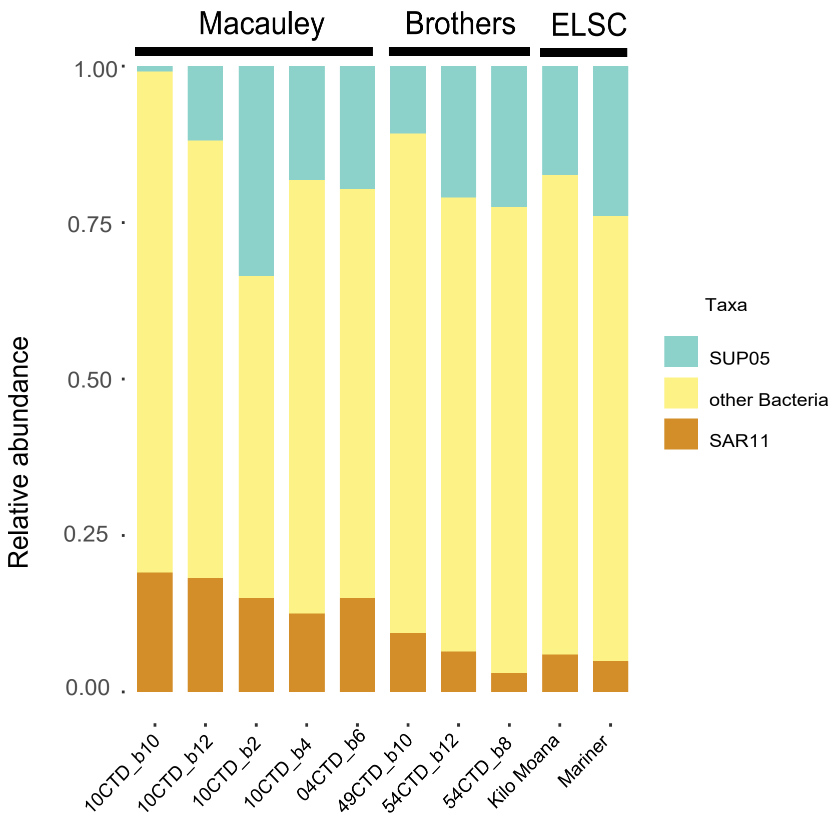


**Figure S8. Taxonomic affiliation of bacterial cytochrome genes across McV, BrV and Mariner and Kilo Moana (Eastern Lau Spreading Centre - ESLC) metagenomes.** The barchart depicts the relative abundance of the cytochrome genes affiliated to SUP05, SAR11, and other Bacteria. Cytochrome genes were analysed with MEGAN 6.6.0 [10] using Lowest Common Ancestor (LCA) assignment algorithm.

| Metagenome assembled genome | Sox | Sqr | Fcc | DsrAB | AprAB | Hydrogenase | CO oxidation | Cytochrome C4 | Cytochrome bc1 | Cytochrome CBB3 | Cytochrome C1 | Cytochrome C2 | Cytochrome CBO3 | Cytochrome C oxidase | rTCA | Reductive acetyl-CoA | CBB | Nitrogenfixation | NAP/NAR | NIR | NOR | NOS | Di-/tricarboxylate transporter | Aminoacid transporter | Dipeptide transporter | Oligopeptide transporter | Long chain fatty acid transporter | Urea transporter | Urease | Urea carboxlase | Flagellum | Twitching type II pilus | Type IV pilus | Chemotaxis |
|---|---|---|---|---|---|---|---|---|---|---|---|---|---|---|---|---|---|---|---|---|---|---|---|---|---|---|---|---|---|---|---|---|---|---|
| uncultured *Chloroflexi* | - | - | - | - | + | - | + | - | - | + | - | + | - | + | + | - | - | - | + | - | - | - | + | + | + | + | + | - | + | + | - | - | - | + |
| uncultured *Acidimicrobiales* | + | - | - | - | - | - | + | + | - | + | - | - | - | + | + | - | - | - | - | + | + | - | + | + | + | + | - | + | + | - | - | + | + | + |
| SAR324 | - | - | - | - | - | - | + | - | + | - | - | - | - | + | + | - | - | - | - | - | - | - | + | + | - | + | + | + | + | - | - | - | - | + |
| Sulfurimonas | + | - | - | - | - | + | - | - | - | + | + | + | - | + | - | - | - | - | - | - | - | - | - | - | + | - | + | - | - | - | + | - | + | + |
| Erythrobacter | - | - | - | - | - | - | + | - | - | + | + | + | - | + | + | - | - | - | - | - | - | - | + | + | - | + | - | + | - | - | + | - | + | + |

**Figure S9. Metabolic potential of non‑SUP05 MAGs with a completeness of >50%.** Bacterial genomes were manually screened for genes for: ‑ sulfur metabolism (sox – sulfur oxidizing enzyme; Sqr – sulfide:quinone oxidoreductase; Fcc – flavocytochrome c; Dsr – dissimilatory sulfite reductase; Apr – dissimilatory adenylylsulfate reductase), hydrogen and carbon monoxide oxidation, carbon fixation pathways (rTCA ‑ reverse tricarboxylic acid cycle, reductive acetyl‑CoA and CBB – Calvin Benson Bassham cycle), nitrogen fixation and nitrogen reduction (NAP – nitrate reductase; NIR – nitrite reductase; NOR – nitric oxide reductase, NOS – nitrous oxide reductase), transporters and lastly motility genes.

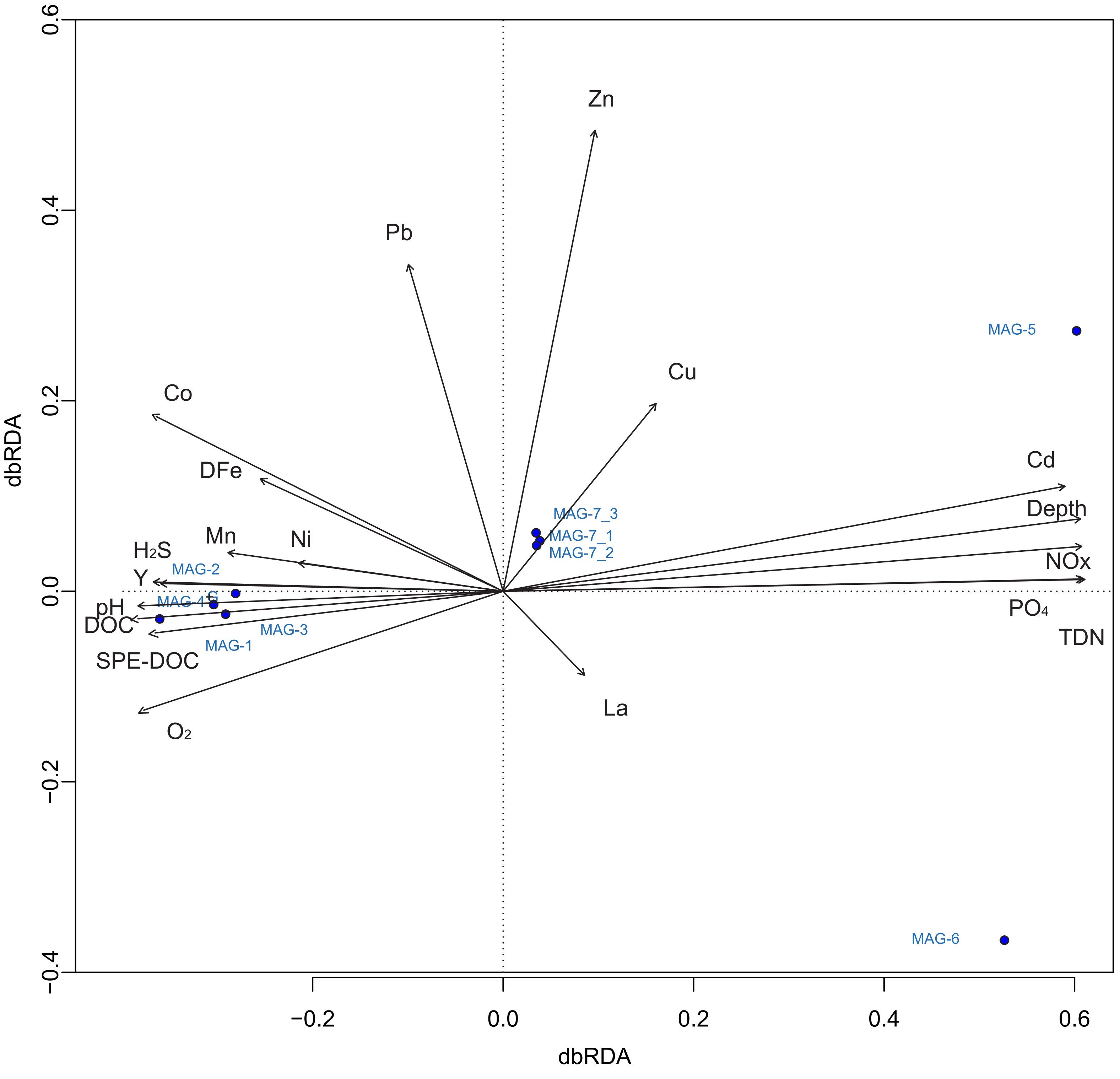


**Figure S10. Distance-based redundancy analysis (dbRDA) of SUP05 MAGs.** This analysis was calculated based on a Bray-Curtis dissimilarity matrix of MAGs' ANI values and standardized log environmental parameters.

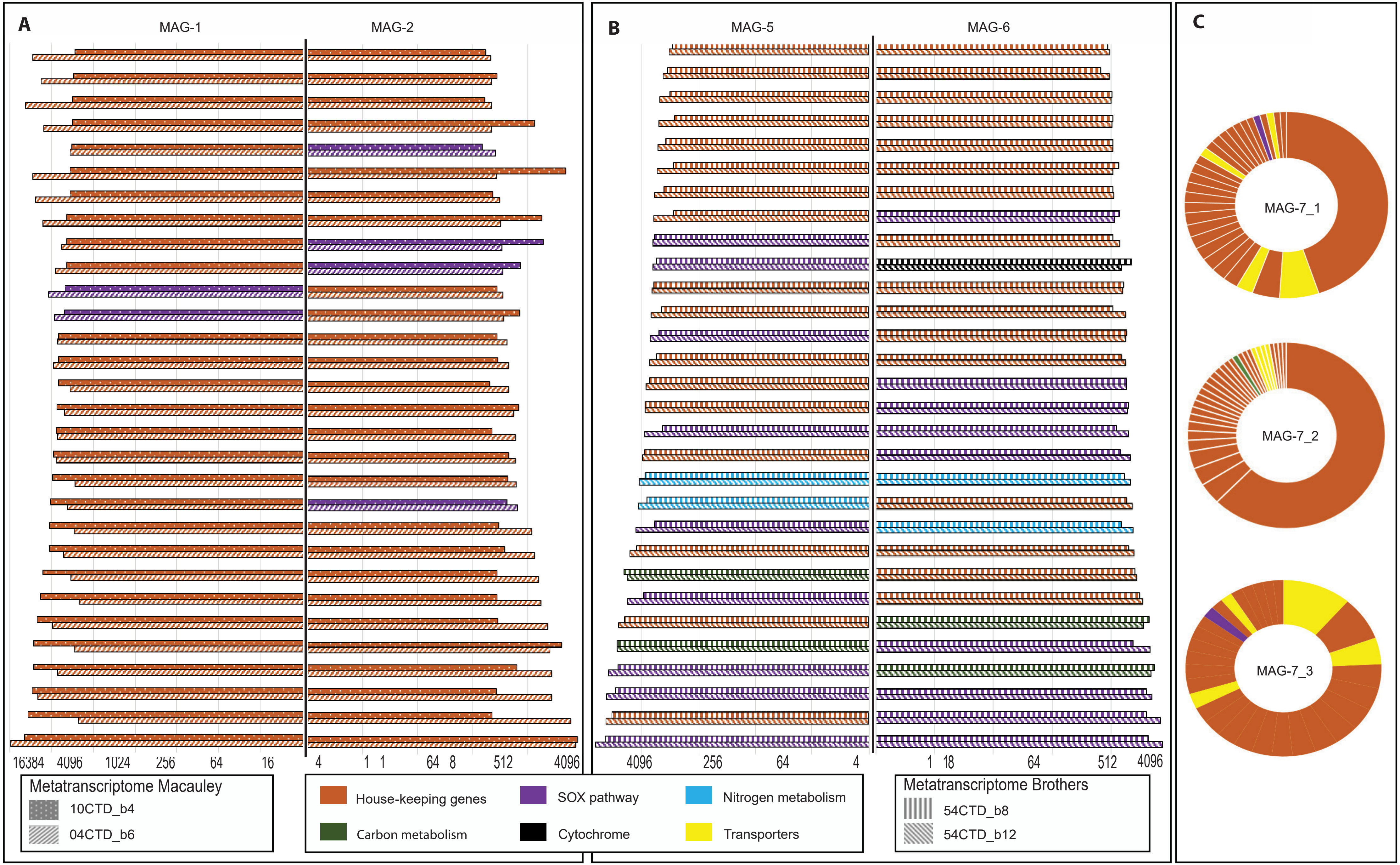


**Figure S11. Relative abundance of the 30 most expressed genes of the most complete SUP05 MAGs (A and B) and putatively heterotrophic MAGs (C).** The relative abundance is given as log normalized transcript per million (TPM). A) Metatranscriptomes of McV were mapped with 97% identity to MAG - 1 and MAG - 2. B) Metatranscriptomes of BrV were mapped (97% identity) to MAG - 5 and MAG - 6. The patterns of the bars link the expression of a specific gene to the metatranscriptome and the coupled-bar represents the same gene in two metatranscriptomes. C) MAG - 7_1 to 3, were mapped (97% identity) to metatranscriptomes of BrV and are given as a sunburst plot. Genes in the SUP05 MAGs, such as house - keeping genes, carbon metabolism, sulfur - oxidation pathway (SOX), cytochromes, nitrogen metabolism and transporters, are represented by different colors.

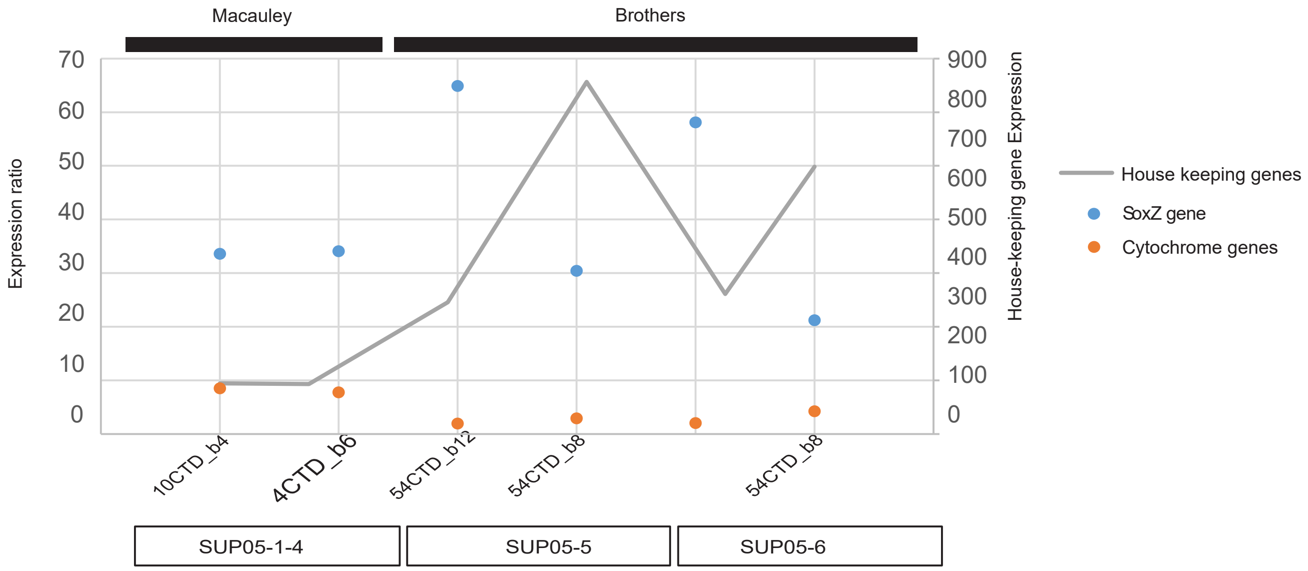


**Figure S12. Expression ratio between cytochrome, sox and house‐keeping genes in six SUP05 MAGs across four metatranscriptomes from McV and BrV.** SUP05‐1‐4 is the average of expression rate in MAG‐1 to MAG‐4. House‐keeping genes analysed are *pro*C, *rec*A and *rpo*D. House‐keeping gene expression is given on the secondary axis for activity comparison between MAGs and metatranscriptomes.

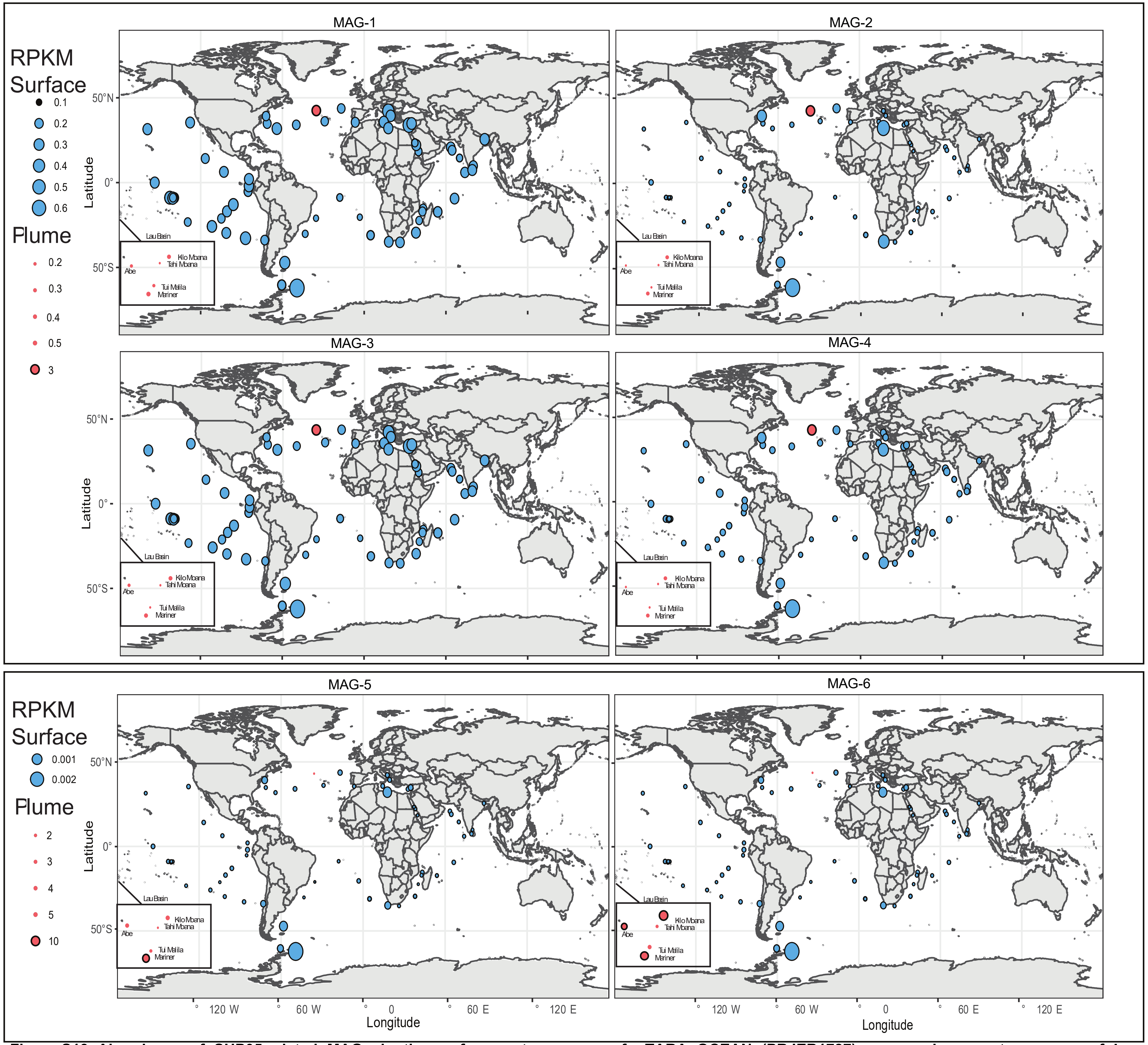


**Figure S13. Abundance of SUP05-related MAGs in the surface metagenomes of TARA OCEAN (PRJEB1787), seven plume metagenomes of Lau Basin (>1900 m depth; Tai Malila, Tahi Moana, Mariner, Kilo Moana, Abe) and Mid-Atlantic ridge (Woody Crack - 828 m depth).** Reads were mapped to the SUP05 clusters using BBmap with 99% minimum identity. Abundance was calculated as RPKM.

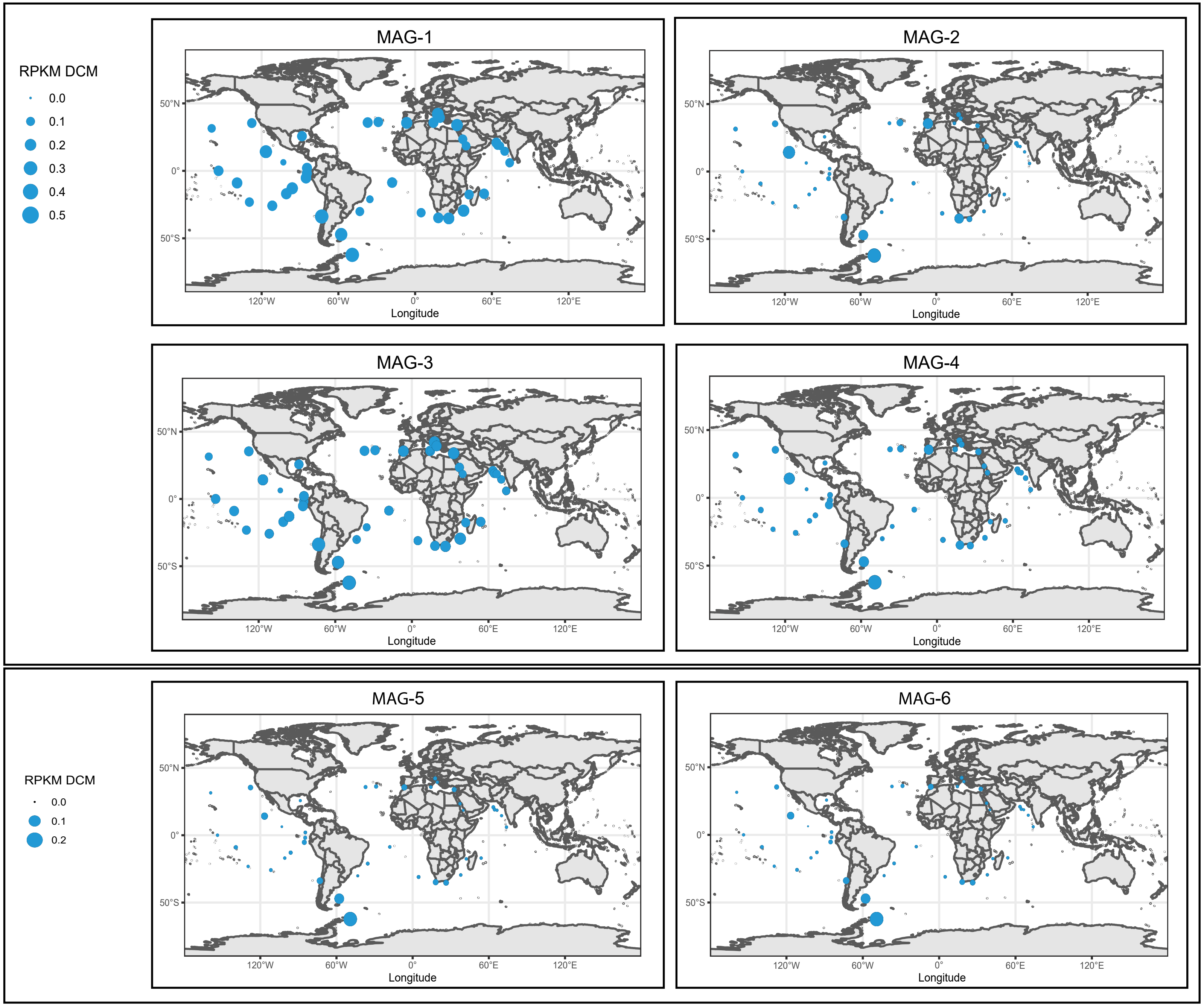


**Figure S14. Abundance of SUP05‑related MAGs in the deep chlorophyll maximum (DCM) metagenomes of TARA Ocean (PRJEB1787).** SUP05 MAG‑1 to 4 are *Candidatus* Thioglobus vadi strains. Reads were mapped unambiguously to the SUP05 clusters using BBmap [27] with 99% minimum identity. Abundance was calculated as RPKM.

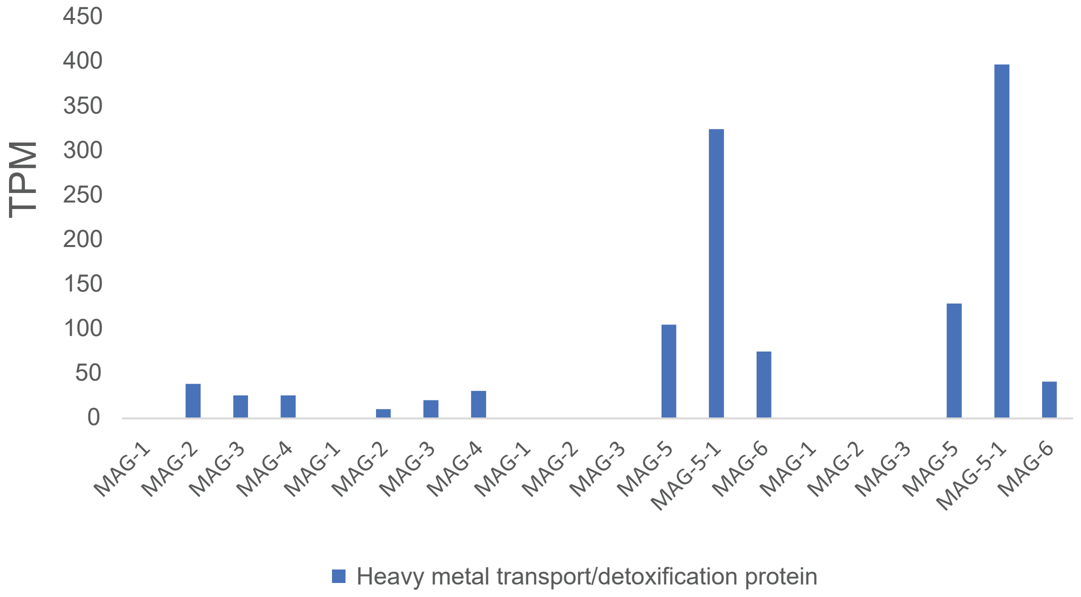


**Figure S15. Expression of genes for heavy-metal transporter/detoxification proteins in SUP05 MAGs.**
